# Neurochemical Pathways That Converge on Thalamic Trigeminovascular Neurons: Potential Substrate for Modulation of Migraine by Sleep, Food Intake, Stress and Anxiety

**DOI:** 10.1371/journal.pone.0103929

**Published:** 2014-08-04

**Authors:** Rodrigo Noseda, Vanessa Kainz, David Borsook, Rami Burstein

**Affiliations:** 1 Department of Anesthesia, Critical Care and Pain Medicine, Beth Israel Deaconess Medical Center, Harvard Medical School, Boston, Massachusetts, United States of America; 2 Department of Anesthesiology, Perioperative and Pain Medicine, Boston Children’s Hospital, Harvard Medical School, Boston, Massachusetts, United States of America; University College London, United Kingdom

## Abstract

Dynamic thalamic regulation of sensory signals allows the cortex to adjust better to rapidly changing behavioral, physiological and environmental demands. To fulfill this role, thalamic neurons must themselves be subjected to constantly changing modulatory inputs that originate in multiple neurochemical pathways involved in autonomic, affective and cognitive functions. Our overall goal is to define an anatomical framework for conceptualizing how a ‘decision’ is made on whether a trigeminovascular thalamic neuron fires, for how long, and at what frequency. To begin answering this question, we determine which neuropeptides/neurotransmitters are in a position to modulate thalamic trigeminovascular neurons. Using a combination of in-vivo single-unit recording, juxtacellular labeling with tetramethylrhodamine dextran (TMR) and in-vitro immunohistochemistry, we found that thalamic trigeminovascular neurons were surrounded by high density of axons containing biomarkers of glutamate, GABA, dopamine and serotonin; moderate density of axons containing noradrenaline and histamine; low density of axons containing orexin and melanin concentrating hormone (MCH); but not axons containing CGRP, serotonin 1D receptor, oxytocin or vasopressin. In the context of migraine, the findings suggest that the transmission of headache-related nociceptive signals from the thalamus to the cortex may be modulated by opposing forces (i.e., facilitatory, inhibitory) that are governed by continuous adjustments needed to keep physiological, behavioral, cognitive and emotional homeostasis.

## Introduction

Historically, the thalamus was viewed as a simple relay station for sensory information from the periphery to the cortex. This view has been replaced by the concept that instead of ‘just’ transferring sensory signals from subcortical nuclei to the cortex, thalamic neurons play central role in the selection, amplification, and prioritization process that determines which type of information should be made available to the cortex at any given time [1], [2]. Being the so-called ‘gate-keeper’ of the cortex, thalamic neurons regulate the flow of rapidly-changing sensory signals, thus allowing the cortex to adjust to the constantly evolving behavioral and environmental demands [1].

To regulate the amount of sensory signals that reach the cortex, thalamic neurons must themselves be subjected to a variety of modulatory inputs that originate in cortical, hypothalamic, brainstem, spinal and intrathalamic nuclei [1], [3]–[6]. In the context of somatosensory and nociceptive information, the more extensively studied networks that drive and/or modulate the activity of relay thalamic neurons are the excitatory glutamatergic input originating in corticothalamic, spinothalamic and medial lemniscus tract neurons, and the inhibitory GABAergic input involving the reticular thalamic nucleus [7], [8]. The excitatory glutamate input, acting through metabotropic mGluRs, is capable of producing sustained neuronal firing whereas the inhibitory GABA input, acting through the GABAb receptor is capable of switching off the sustained neuronal activity [8].

Far less is known about the regulation of relay thalamic neurons by other neurotransmitters and neuropeptides [3] from various brain regions. Candidates include those from the brainstem and hypothalamus. Brainstem inputs include serotonergic projections from raphe nuclei [9], [10], noradrenergic projections from locus coeruleus and the A5 catecholamine group in the pons [9]–[11], and dopaminergic projections from periaqueductal gray, and the lateral parabrachial nucleus [12]–[16]. Hypothalamic inputs include additional dopaminergic projections from A11/A13 [12]–[16], histaminergic projections from the tuberomammillary nucleus [17], [18], orexinergic projections from the perifornical, dorsomedial and lateral hypothalamus [19], [20], and melanin-concentrating hormone (MCH) projections from the lateral hypothalamus [21]–[23].

The potential release of these neurotransmitters/neuropeptides on relay thalamic nuclei suggests that the modulation of individual neurons is rather complex, likely subjected to opposing forces driven by a variety of changing external and internal conditions that require constant behavioral, physiological, and affective adjustments. Our overall goal is to understand how ‘a decision’ is made on whether or not a relay thalamic neuron fires, for how long, and at what frequency. To start answering this question, we must first determine which neuropeptides/neurotransmitters are in a position to govern the activity of individual thalamic neurons that share a common function; a task never taken before. In the current study we describe an array of neuropeptides/neurotransmitters that may modulate individual, physiologically-identified thalamic trigeminovascular neurons believed to play a role in the generation of headache perception during migraine. The understanding of this neurobiology will allow for a basis to determine functional neurotransmission between the thalamus and cortex related to multiple clinical components of migraine including pain (somatosensory cortex), cognition (frontal cortex), memory (hippocampus), altered perception (parietal cortex), interoception and awareness (insular cortex).

## Materials and Methods

### Animal preparation

Experiments were approved by the Institutional Animal Care and Use Committee at Harvard Medical School and Beth Israel Deaconess Medical Center, and conducted in accordance of NIH guide for the care and use of laboratory animals. Thirty-two male Sprague-Dawley rats weighing 250–350 g were initially anesthetized with a single dose of Brevital sodium (45 mg/kg i.p.) to allow endotracheal intubation and cannulation of the right femoral vein. Each rat was then mounted on a stereotaxic frame and connected to an inhalation anesthesia system (O^2^/Isoflurane 2.5% for craniotomies; 1–1.2% for maintenance during the rest of the experiment, delivered at 100 ml/min). End-tidal CO_2_, respiratory and heart rate, blood oxygen saturation and body temperature were continuously monitored and kept within a physiological range. One craniotomy was performed at the left lambdoid suture to expose and stimulate the meninges overlying the left transverse sinus. A second craniotomy was performed at the right parietal bone to allow the introduction of a glass micropipette into the posterior thalamus for recording and juxtacellular iontophoresis of an anterograde tracer, as described previously [24]. The exposed dura was kept moist throughout the experiment, using synthetic interstitial fluid (SIF; pH 7.2). After surgery, a lactated ringer’s solution with a mixture of paralytic agents (vecuronium/doxacurium) was continuously infused via the femoral vein cannula (0.25 mg/kg/hr).

### Single-unit juxtacellular recording and iontophoresis

**Figure 1:**
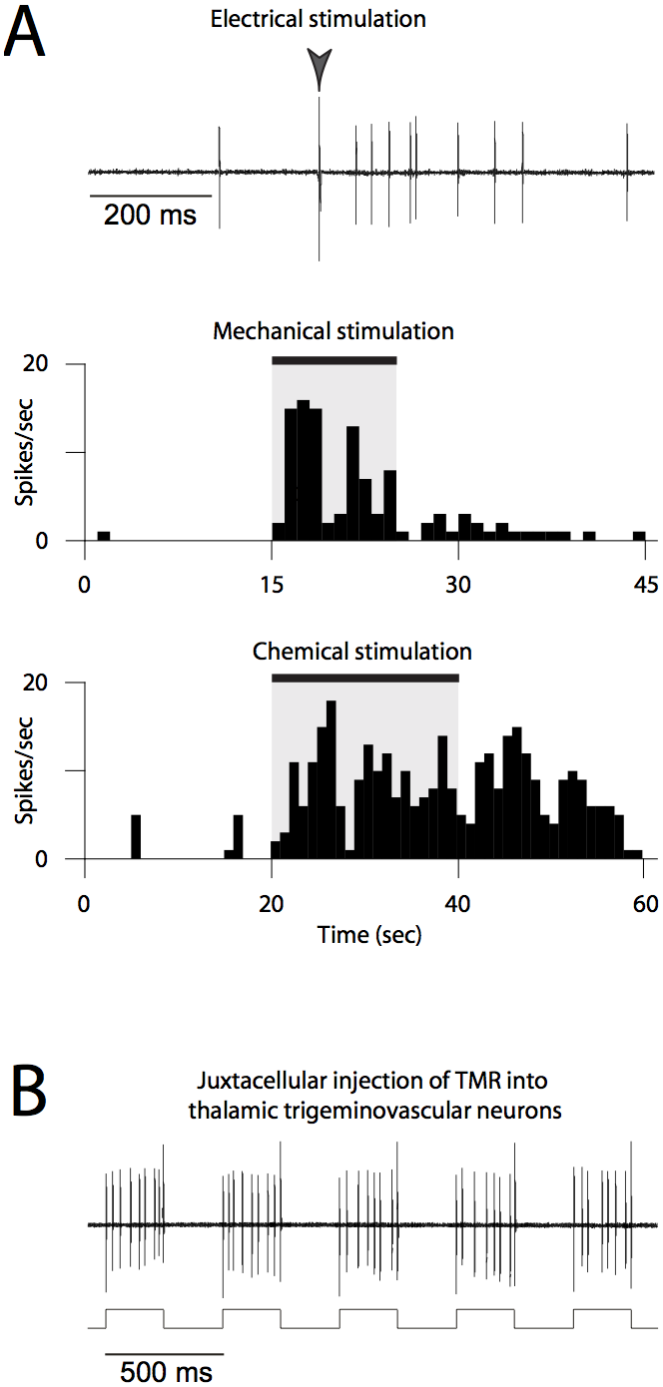
Identification and labeling of individual thalamic trigeminovascular neurons. (A) Neuronal responses to electrical (1 mA, 0.8 ms), mechanical (von Frey filament: 4, 63 g) and chemical (1 M KCl solution) stimulation of the dura overlying the left transverse sinus. (B) Synchronization of neuronal activity during iontophoretic injection of TMR by delivering pulses of current (1–10 nA) at 250 ms on/off intervals through the recording glass micropipette.

A glass micropipette (20–30 MΩ impedance) filled with a 3% solution of the tracer tetramethylrhodamine dextran (TMR; 3,000 MW, anionic, lysine fixable; D-3308, Invitrogen) in 0.9% NaCl, was lowered into the right posterior thalamus while searching for single-unit responses to electrical stimulation of the contralateral dura (0.8 ms, 0.5–3.0 mA, 1 Hz). Thalamic neurons responding to the electrical stimulation were additionally tested for responses induced by mechanical (calibrated von-Frey monofilament) and chemical (1 M KCl) stimulation of the dura (Fig. 1A). Response was defined as an increase in firing rate that was at least 50% higher than baseline. Spikes from neurons responding to all three types of stimuli were amplified, filtered and acquired in a window discriminator to be further analyzed using Spike2 software (CED, Cambridge, UK). Once the electrophysiological characterization of neuronal responses was finalized, the cell was iontophoretically injected using the recording glass micropipette by delivering pulses of positive current (1–10 nA) at 250-ms on/off intervals by means of a computer-controlled microelectrode amplifier (Axoclamp 900A, Molecular Devices), as described elsewhere [25] (Fig. 1B). In some experiments, more than one injection was performed in different locations of the posterior thalamus. After a period of 10–20 min of juxtacellular filling, the micropipette was slowly pulled out of the brain; the animal remained anesthetized for 30 minutes and then was prepared for perfusion.

### Histological processing

Rats were injected with an overdose of pentobarbital sodium (100 mg/kg) and perfused intracardially with 200 ml heparinized saline, followed by a fixative solution consisting in 400 ml of 0.1 M phosphate buffered saline (PBS), 4% paraformaldehyde and 0.05% picric acid. Only when required for the staining protocol, rats were perfused with 200 ml of PBS followed by a fixative solution containing 75 ml of 4% ethylcarbodiimide in 0.1 M PBS. Brains were removed, soaked in the fixative solution for 2 hrs, and cryoprotected in 30% sucrose phosphate buffer for 48 hrs. Brains were then frozen and cut into serial coronal sections (60–80 µm-thick) using a cryostat (Leica). Free-floating sections were collected and mounted on slides for a rapid visualization and localization of each cell body and its dendrites using epifluorescence microscopy.

### Immunofluorescence

Brain sections containing successfully injected neurons were pre-incubated at room temperature in PBS containing 2% fetal bovine serum albumin (FSA) and 1% Triton X-100 for 1 hr. Sections were then incubated at 4°C for 48 hrs in the same blocking solution with one of the following primary antibodies: (i) mouse anti-Serotonin Transporter, SERT (1∶5,000 dilution; Millipore); (ii) mouse anti-Tyrosine Hydroxylase, TH (1∶5,000; Immunostar); (iii) rabbit anti-Dopamine β-Hydroxylase, DBH (1∶5,000; Immunostar); (iv) goat anti-Orexin A (1∶2,500; Santa Cruz); (v) rabbit anti-Calcitonin Gene Related Peptide, CGRP (1∶5,000; Chemicon); (vi) rabbit anti-5HT_1D_ receptor (1∶50,000; Courtesy of Andrew Ahn, University of Florida); (vii) rabbit anti-Oxytocin (1∶10,000; Immunostar); (viii) goat anti-Vasopressin (1∶1,000; Immunostar); (ix) rabbit anti-Histamine (1∶3,000; Immunostar; ethylcarbodiimide perfusion); (x) guinea pig anti-Vesicular Glutamate Transporter 2, VGluT2 (1∶2,500; Millipore); (xi) rabbit anti-Vesicular GABA Transporter, VGaT (1∶1,000; Phosphosolutions); (xii) Melanin Concentrating Hormone, MCH (1∶1,000; Courtesy of Terry Maratos-Flier, Harvard Medical School). The sections were washed multiple times and then incubated in PBS containing 2% FSA and 0.5% Triton X-100 for 2 hrs at room temperature with the corresponding fluorescent secondary antibody (Alexa Fluor 488; Invitrogen) against the Ig’s of the animal in which the primary antibody was raised (dilution range 1∶200–1∶1,000). Immunostained sections were serially mounted on glass slides and coverslipped with fluorescent mounting media with or without DAPI counterstaining.

### Digital imaging of thalamic labeling

Digital imaging of each of the neuronal cell bodies and dendrites injected with TMR, as well as the axonal network of the different neurotransmitters/neuropeptides was performed using epifluorescence scanning microscopy that compiled 1–1.5 µm-thick scans using *z*-stacking software (Leica). Using individual z-stack images, orthogonal views of the y–z and x–z planes were also created to provide additional evidence for close apposition, and thus probable contact. Immunofluorescent labeling of TMR was detected by excitation/emission at 551/624 nm (red). For the axonal labeling of neurotransmitters/neuropeptides with Alexa Fluor 488, the immunolabeling was detected by excitation/emission at 455/520 nm (green). DAPI counterstaining was detected by excitation/emission at 358/461 nm (blue). Co-labeling of the different structures was achieved by superimposition of the red, green and blue images. The anatomical analysis and localization of cell bodies was based on a rat brain atlas [26]. Quantitative measures were performed to obtain the relative innervation density of neurotransmitters/neuropeptides in the thalamic regions of interest. A qualitative approach was used to determine close apposition between immunopositive axons and thalamic trigeminovascular neurons.

## Results

### Identification and juxtacellular labeling of thalamic trigeminovascular neurons

Forty-seven thalamic neurons that responded to electrical, mechanical and chemical stimulation of the contralateral dura were classified as trigeminovascular neurons [24]. Twenty-four of these neurons were successfully injected with TMR, yielding a detailed, high-resolution labeling of the cell body (seen in 1 or 2 sections), dendritic tree and proximal segment of the parent axon (extending over 5–6 sections) within the thalamus. In each of these cases, attempts were made to co-label the brain sections containing the TMR-positive trigeminovascular neurons with a different neuropeptide/neurotransmitter. Eighteen cases yielded successful labeling of both trigeminovascular neurons (TMR) and axons stained for markers of glutamate (n = 2), GABA (n = 2), serotonin (n = 3), noradrenalin (n = 2), dopamine (n = 3), histamine (n = 2), orexin (n = 2), MCH (n = 2), CGRP (n = 2), 5HT_1D_ receptor (n = 2), oxytocin (n = 1) and vasopressin (n = 1).

### Innervation of thalamic trigeminovascular neurons by the spinal trigeminal (SpV) and the reticular thalamic nucleus (Rt)

#### Glutamatergic innervations

**Figure 2:**
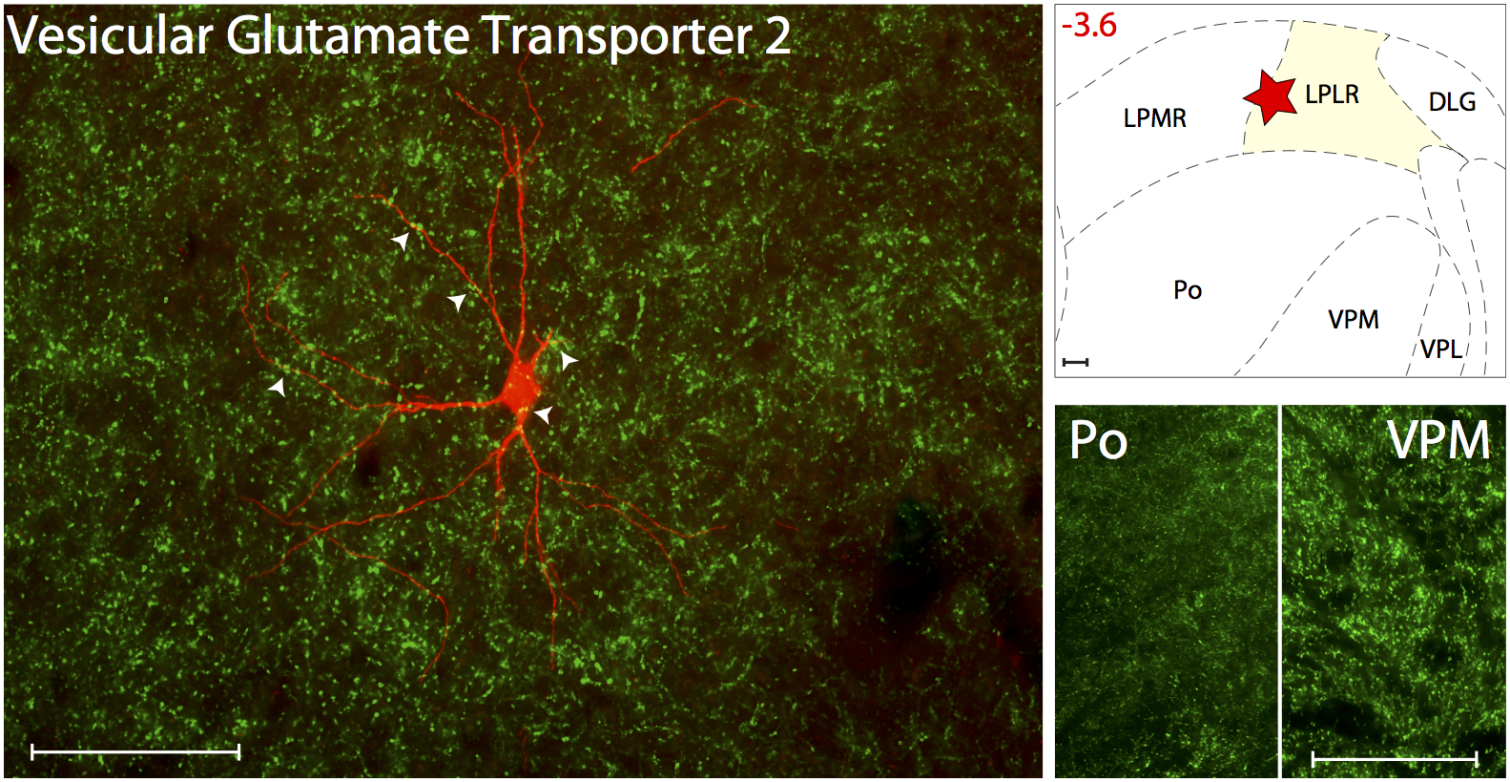
Glutamatergic innervation of thalamic trigeminovascular neurons. Left: Immunopositive VGluT2 synaptic vesicles (green) surrounding a thalamic dura-sensitive neuron (red) labeled with TMR–dextran. Arrowheads indicate close apposition of VGluT2 positive axons and the cell body and dendrites of the labeled neuron. Upper right: Location of the dura-sensitive neuron (red star) shown at left. Number in red indicates distance from *bregma* (mm). Lower right: Fluorescent images showing VGluT2 axonal labeling in thalamic Po and VPM nuclei. Scale bars = 100 µm. Abbreviations: DLG, dorsal lateral geniculate; LPMR, lateral posterior thalamic, mediorostral; LPLR, lateral posterior thalamic, laterorostral part; VPL, ventral posterolateral thalamic.

Glutamatergic innervation was determined using Vesicular Glutamate Transporter 2 (Fig. 2). Axons immunoreactive to VGluT2, thus containing the excitatory neurotransmitter glutamate, were present at high density in all thalamic nuclei known to contain trigeminovascular neurons including ventral posteromedial (VPM), posterior (Po), lateral posterior (LP) and laterodorsal (LD). When examined in sections containing the trigeminovascular neuron(s), dense VGluT2 immunopositive vesicles were seen in close apposition to the cell body, proximal and distal dendrites (Fig. S1).

#### GABAergic innervations

**Figure 3:**
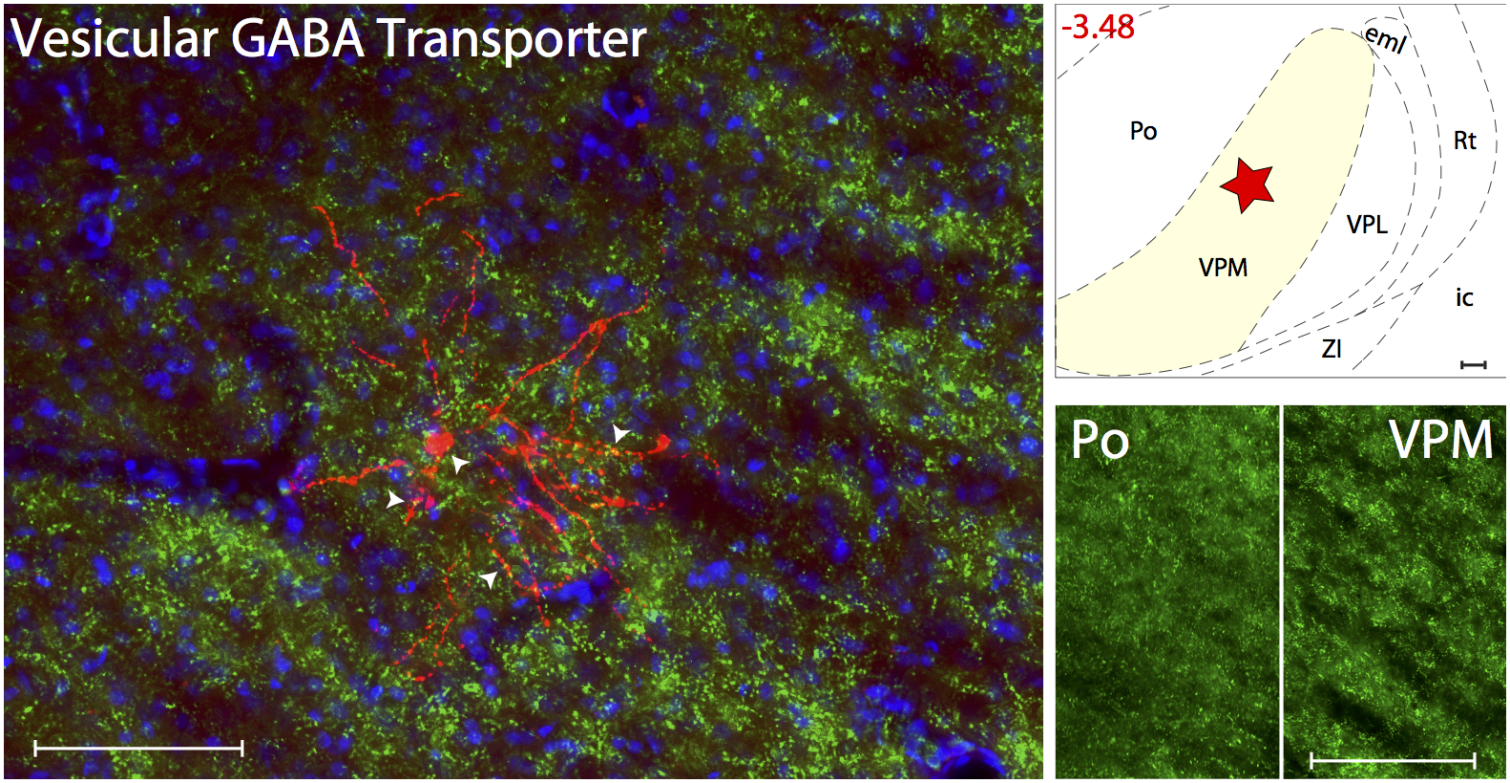
GABAergic innervation of thalamic trigeminovascular neurons. Left: Immunopositive VGaT synaptic vesicles (green) surrounding a thalamic dura-sensitive neuron (red) labeled with TMR–dextran. Nuclear counterstaining was performed with DAPI (blue). Arrowheads indicate close apposition of VGaT positive axons and the cell body and dendrites of the labeled neuron. Upper right: Location of the dura-sensitive neuron (red star) shown at left. Number in red indicates distance from *bregma* (mm). Lower right: Fluorescent images showing VGaT axonal labeling in thalamic Po and VPM nuclei. Scale bars = 100 µm. Abbreviations: eml, external medullary lamina; ic, internal capsule; ZI, zona incerta.

GABAergic innervation was determined using Vesicular GABA Transporter (Fig. 3). Axons immunoreactive to VGaT, thus containing the inhibitory neurotransmitter GABA, were present at high density in all thalamic nuclei known to contain trigeminovascular neurons. When examined in sections containing the trigeminovascular neuron(s), dense VGaT immunopositive vesicles were seen in close apposition to the cell body, proximal and distal dendrites (Fig. S2).

### Brainstem innervation of thalamic trigeminovascular neurons

#### Serotoninergic innervations

**Figure 4:**
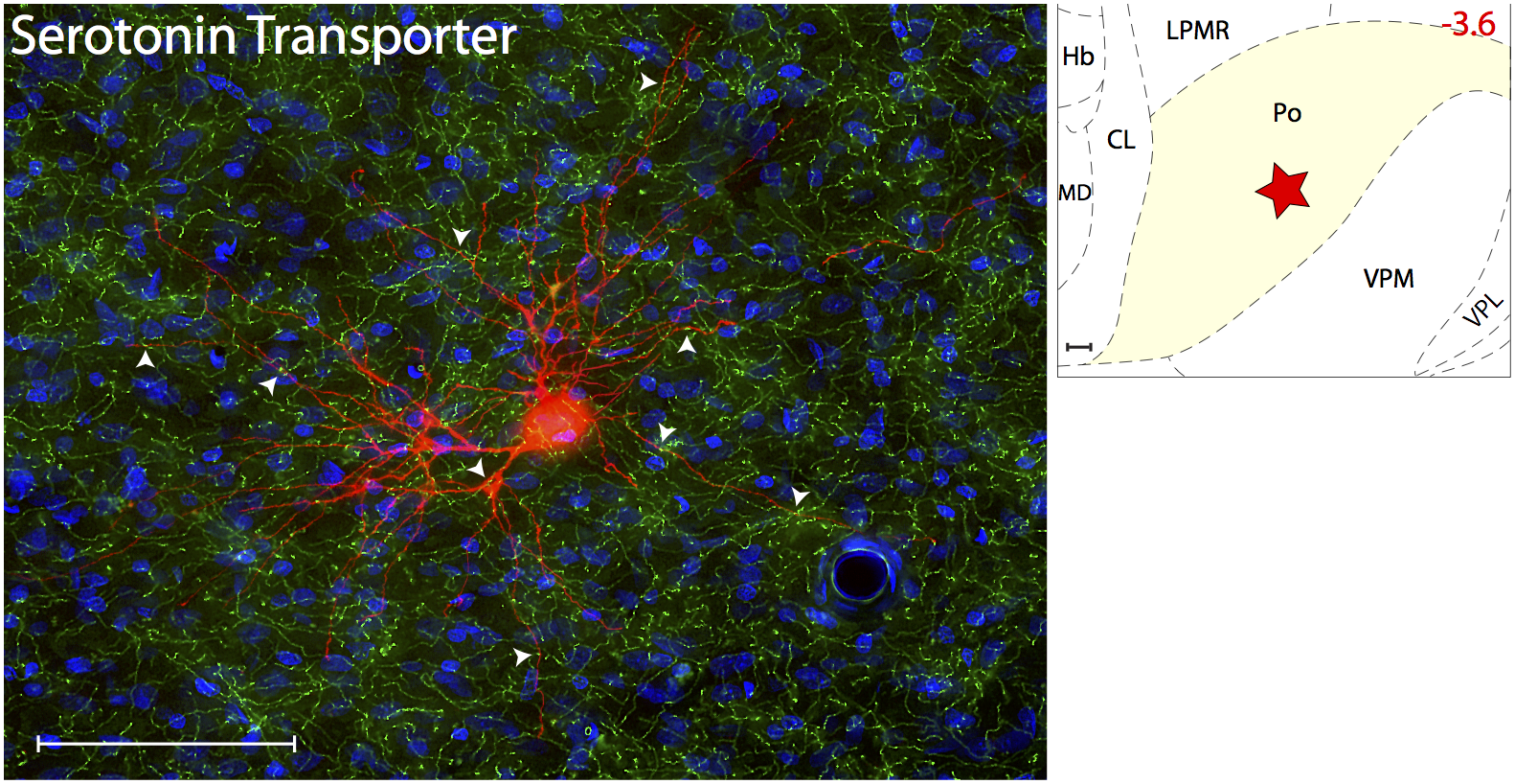
Serotoninergic innervation of thalamic trigeminovascular neurons. Left: Immunopositive Serotonin Transporter axons (green) surrounding a thalamic dura-sensitive neuron (red) labeled with TMR–dextran. Nuclear counterstaining was performed with DAPI (blue). Arrowheads indicate close apposition of SERT positive axons and the cell body and dendrites of the labeled neuron. Upper right: Location of the dura-sensitive neuron (red star) shown at left. Number in red indicates distance from *bregma* (mm). Scale bars = 100 µm. Since SERT does not stain cell somas, it was not possible to use this marker to identify the serotoninergic neurons in the raphe nuclei that project to the thalamic nuclei containing trigeminovascular neurons. Abbreviations: Hb, habenula; MD, mediodorsal thalamic; CL, centrolateral thalamic.

**Figure 5:**
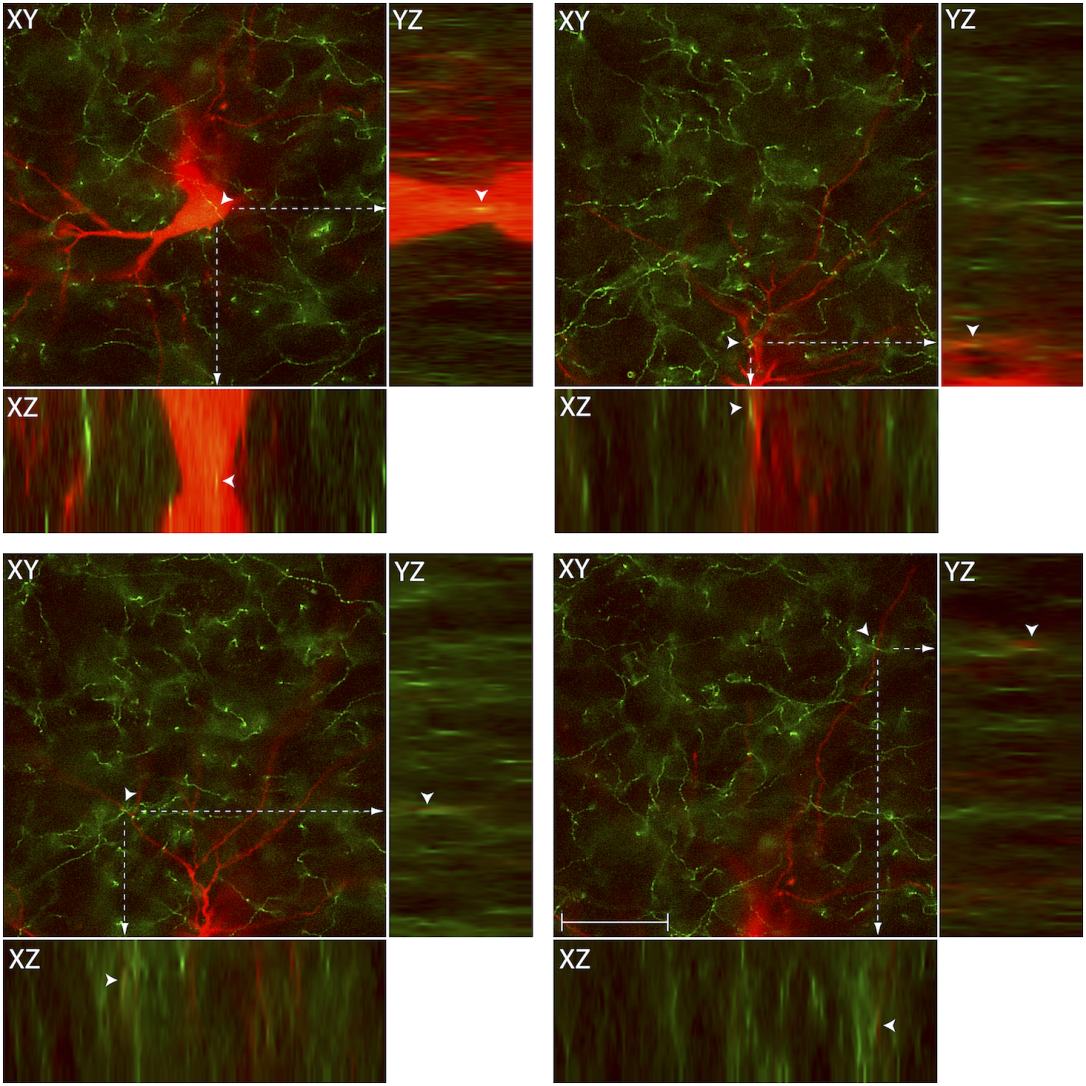
Close apposition between chemically-identified axons and thalamic trigeminovascular neurons. Images from the original z-stack (obtained every 1 µm) were used to create orthogonal views in the y–z and x–z planes. The three views provide evidence that SERT immunopositive fibers (green) may contact cell bodies, proximal and distal dendrites of trigeminovascular neurons in Po (red; as shown in Fig. 4). Note that some green-labeled axons and red-labeled soma or dendrites are in the same focal plane (yellow). To see similar images for all the neurotransmitters and neuropeptides identified in this study, see Supplementary Figures 1–7. *Caveat*: proximity between the chemically-identified axons and the TMR-labeled trigeminovascular thalamic neurons suggests that they are innervated by the different neuropeptides/neurotransmitters. Definitive evidence for actual synapses, however, requires tissue examination with electron microscopy. Scale bar = 50 µm.

Serotoninergic innervation was determined using Serotonin Transporter (Fig. 4), a stable marker of serotoninergic fibers in the brain [27]. Axons immunoreactive to SERT, thus containing the monoamine neurotransmitter serotonin, were present at high density in all thalamic nuclei known to contain trigeminovascular neurons. When examined in sections containing the trigeminovascular neuron(s), dense SERT immunopositive axons and varicosities were seen in close apposition to the cell body, proximal and distal dendrites (Fig. 5).

#### Noradrenergic innervations

**Figure 6:**
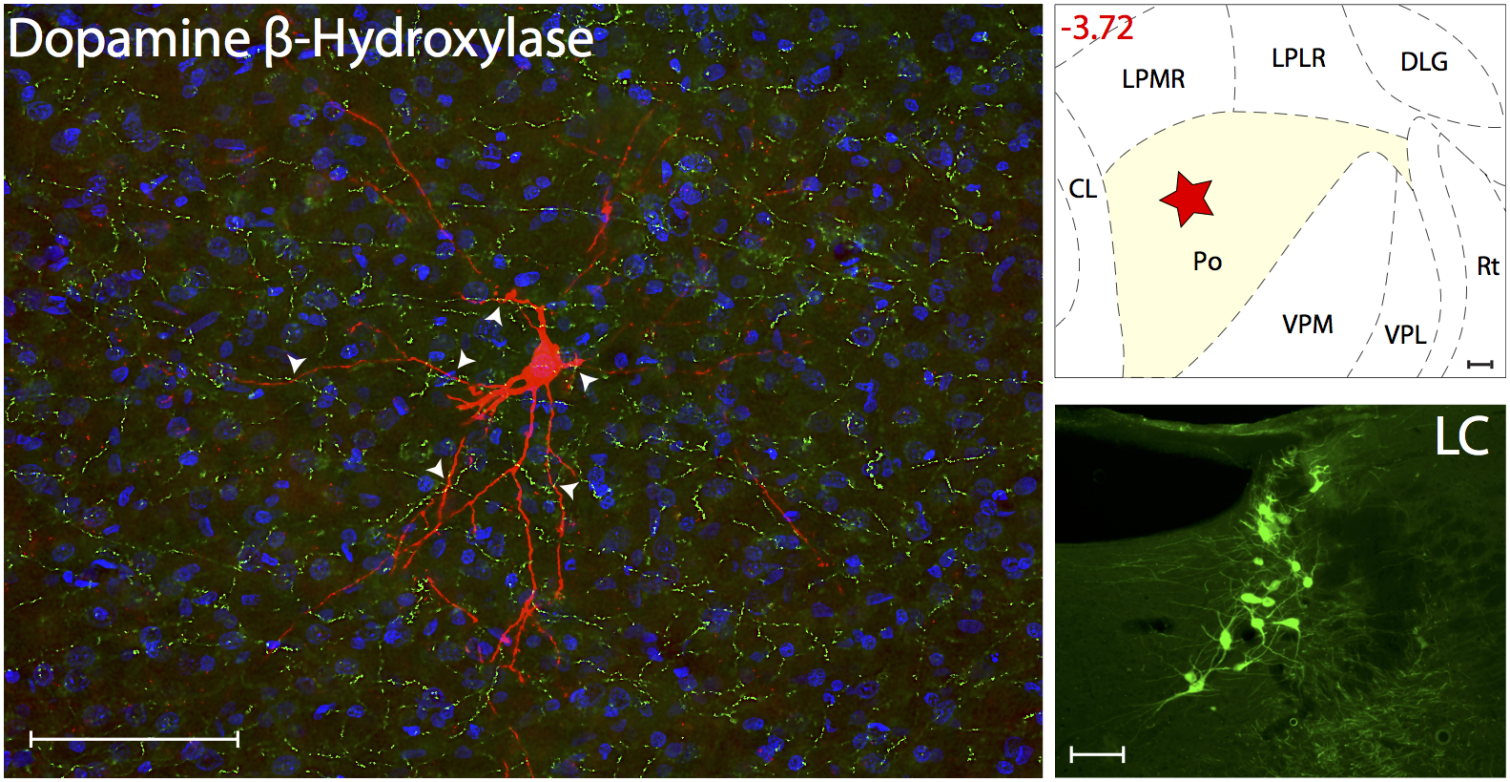
Noradrenergic innervation of thalamic trigeminovascular neurons. Left: Immunopositive Dopamine β-Hydroxylase axons (green) surrounding a thalamic dura-sensitive neuron (red) labeled with TMR–dextran. Nuclear counterstaining was performed with DAPI (blue). Arrowheads indicate close apposition of DBH positive axons and the cell body and dendrites of the labeled neuron. Upper right: Location of the dura-sensitive neuron (red star) shown at left. Number in red indicates distance from *bregma* (mm). Lower right: Fluorescent image showing DBH labeling of cell bodies in the locus coeruleus (LC) of the brainstem. Scale bars = 100 µm.

Noradrenergic innervation was determined using the enzyme Dopamine β-Hydroxylase (Fig. 6). Axons immunoreactive to DBH, thus containing the catecholamine neurotransmitter noradrenaline, were present at moderate-to-high density in all thalamic nuclei known to contain trigeminovascular neurons. When examined in sections containing the trigeminovascular neuron(s), moderate density of DBH immunopositive axons and varicosities were seen in close apposition to the cell body, proximal and distal dendrites (Fig. S3). These DBH axons originate in the locus coeruleus, the main producer of noradrenalin in the brain.

### Hypothalamic innervation of thalamic trigeminovascular neurons

#### Dopaminergic innervations

**Figure 7:**
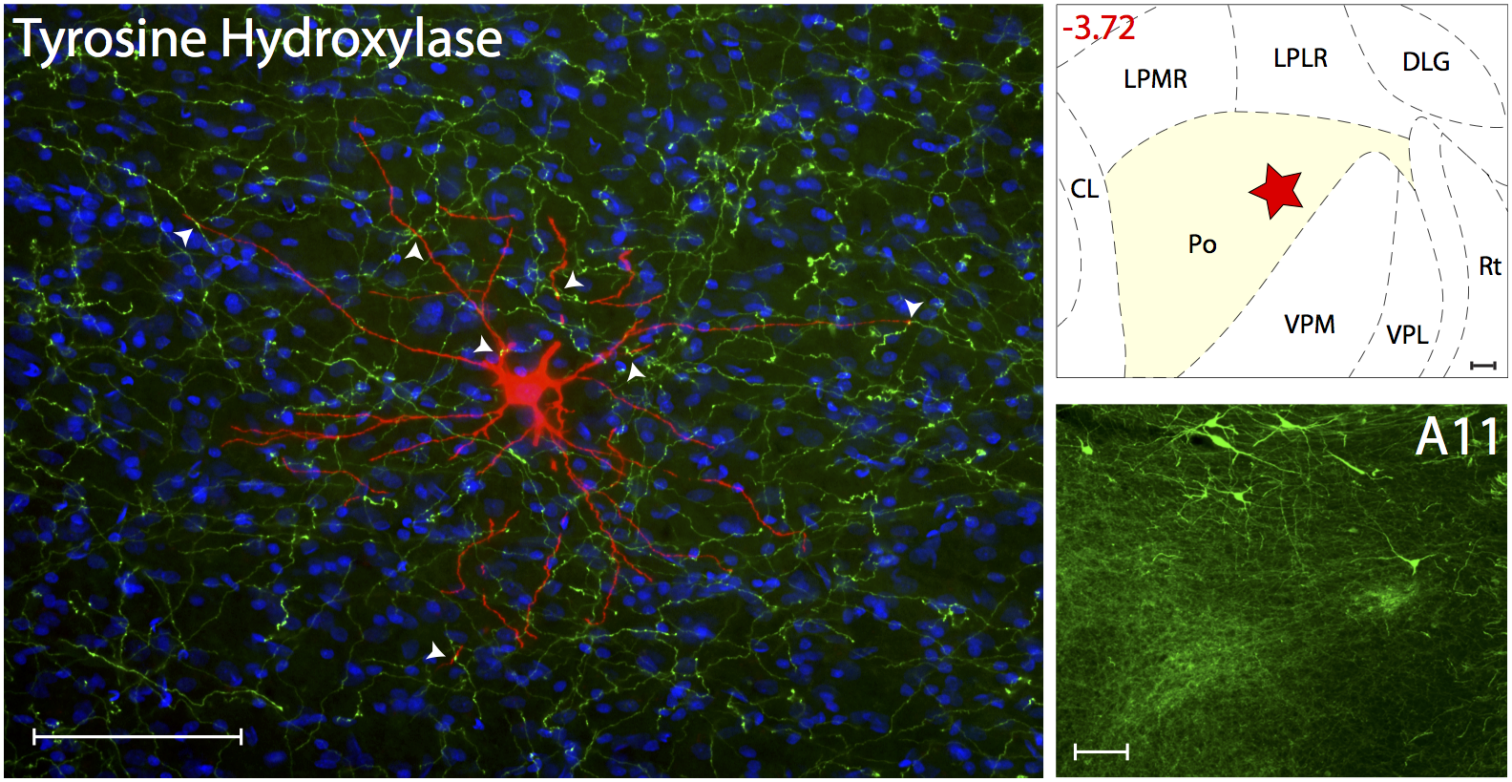
Dopaminergic innervation of thalamic trigeminovascular neurons. Left: Immunopositive Tyrosine Hydroxylase axons (green) surrounding a thalamic dura-sensitive neuron (red) labeled with TMR–dextran. Nuclear counterstaining was performed with DAPI (blue). Arrowheads indicate close apposition of TH positive axons and the cell body and dendrites of the labeled neuron. Upper right: Location of the dura-sensitive neuron (red star) shown at left. Number in red indicates distance from *bregma* (mm). Lower right: Fluorescent image showing TH labeling of cell bodies in the hypothalamic A11 nucleus. Scale bars = 100 mm. *Caveat:* TH is present in noradrenergic and dopaminergic cells, thus TH-positive labeling must take into consideration these two neurotransmitters.

Dopaminergic innervation was determined using the enzyme Tyrosine Hydroxylase (Fig. 7). Axons immunoreactive to TH, thus containing the catecholamine neurotransmitter dopamine, were present at high density in all thalamic nuclei known to contain trigeminovascular neurons. When examined in sections containing the trigeminovascular neuron(s), high density of TH immunopositive axons and varicosities were seen in close apposition to proximal and distal dendrites (Fig. S4). Because TH is present in noradrenergic and dopaminergic cells, the interpretation of its labeling must take into consideration these two neurotransmitters. We interpreted some of the TH-positive axons as dopaminergic based on a recent retrograde tracing study where we showed that the dopaminergic cells group A11/A13 project to the same Po and LP areas in which trigeminovascular neurons were labeled in the current study [28].

#### Histaminergic innervations

**Figure 8:**
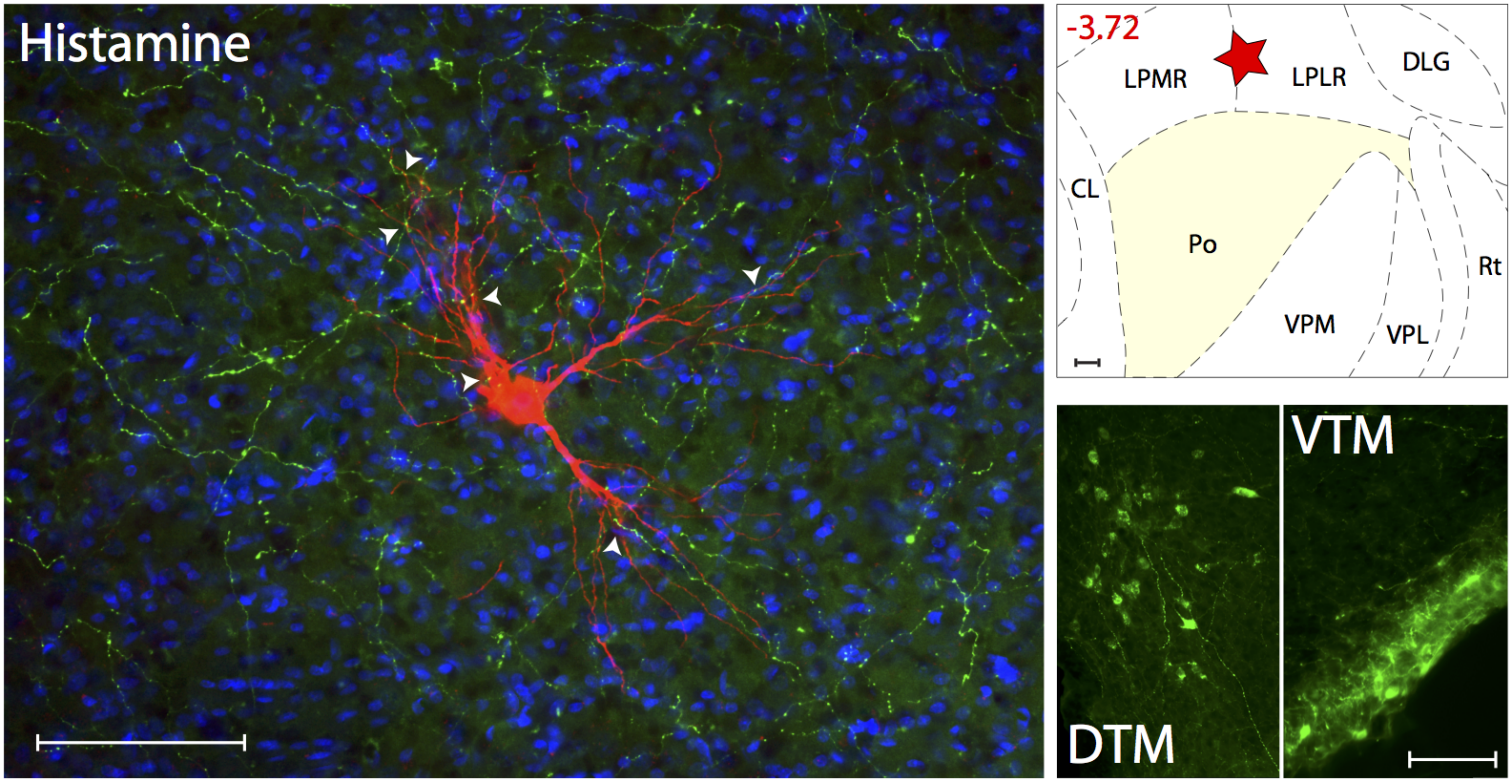
Histaminergic innervation of thalamic trigeminovascular neurons. Left: Immunopositive Histamine axons (green) surrounding a thalamic dura-sensitive neuron (red) labeled with TMR–dextran. Nuclear counterstaining was performed with DAPI (blue). Arrowheads indicate close apposition of Histamine positive axons and the cell body and dendrites of the labeled neuron. Upper right: Location of the dura-sensitive neuron (red star) shown at left. Number in red indicates distance from *bregma* (mm). Lower right: Fluorescent image showing Histamine labeling of cell bodies in the dorsal and ventral tuberomammillary nuclei of the hypothalamus (DTM and VTM). Scale bars = 100 µm.

(Fig. 8). Axons immunoreactive to histamine neurotransmitter were present at moderate density in LP and LD, and lower density in VPM and Po. When examined in sections containing the trigeminovascular neuron(s), moderate density of histaminergic immunopositive axons and varicosities were seen in close apposition to the cell body, proximal and distal dendrites (Fig. S5). This histaminergic innervation originates in the dorsal and ventral tuberomammillary nuclei of the hypothalamus, the sole producers of histamine in the brain.

#### MCH innervations

**Figure 9:**
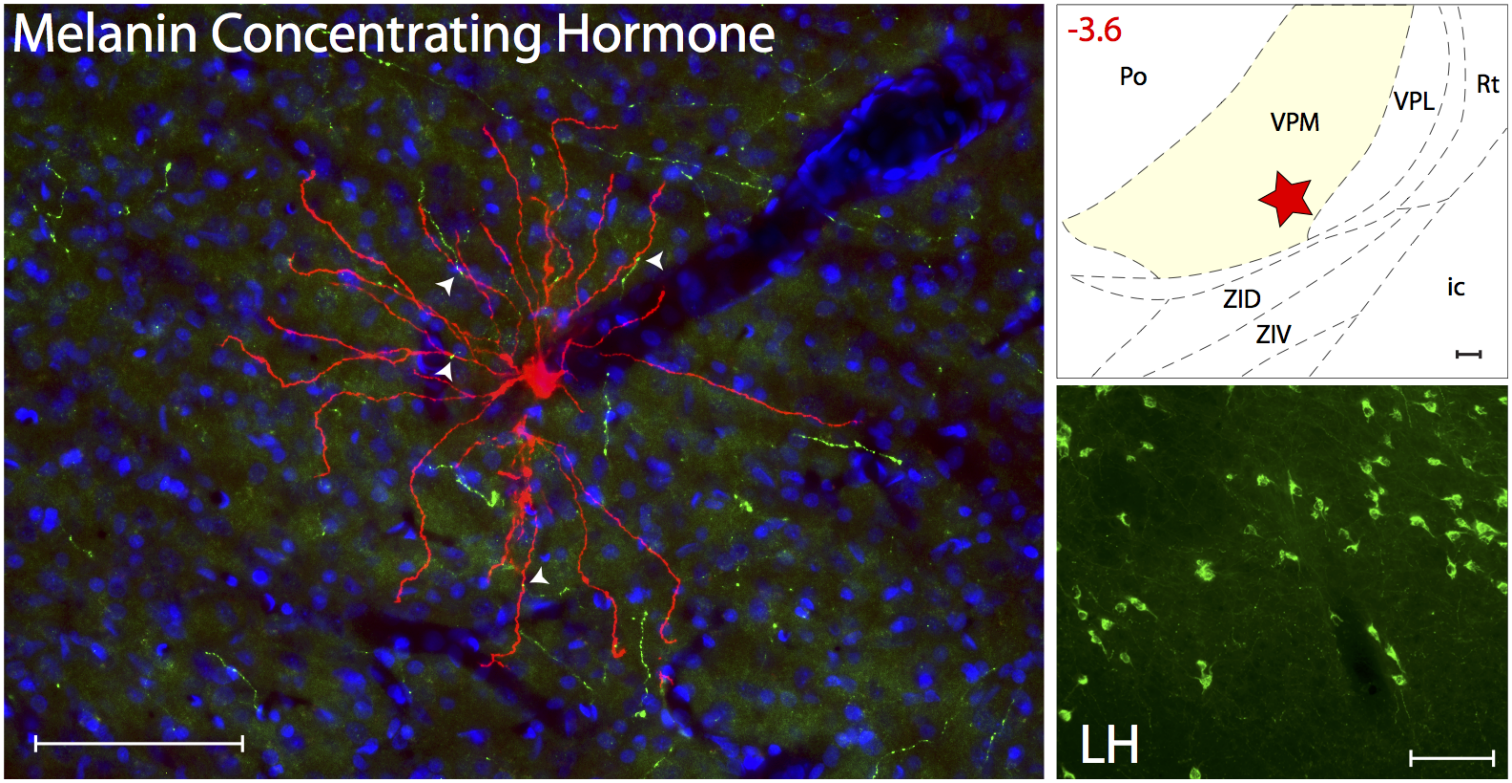
MCH innervation of thalamic trigeminovascular neurons. Left: Immunopositive Melanin Concentrating Hormone axons (green) surrounding a thalamic dura-sensitive neuron (red) labeled with TMR–dextran. Nuclear counterstaining was performed with DAPI (blue). Arrowheads indicate close apposition of MCH positive axons and the dendrites of the labeled neuron. Upper right: Location of the dura-sensitive neuron (red star) shown at left. Number in red indicates distance from *bregma* (mm). Lower right: Fluorescent image showing MCH labeling of cell bodies in the lateral hypothalamus (LH). Scale bars = 100 µm. Abbreviations: ZID, zona incerta, dorsal; ZIV, zona incerta, ventral.

*(* Fig. 9) Axons immunoreactive to MCH were present at low density in all thalamic nuclei known to contain trigeminovascular neurons. When examined in sections containing the trigeminovascular neuron(s), low density of MCH immunopositive axons and varicosities were seen in close apposition only to distal dendrites (Fig. S6). These MCH axons originate mainly in the lateral hypothalamus.

#### Orexinergic innervations

**Figure 10:**
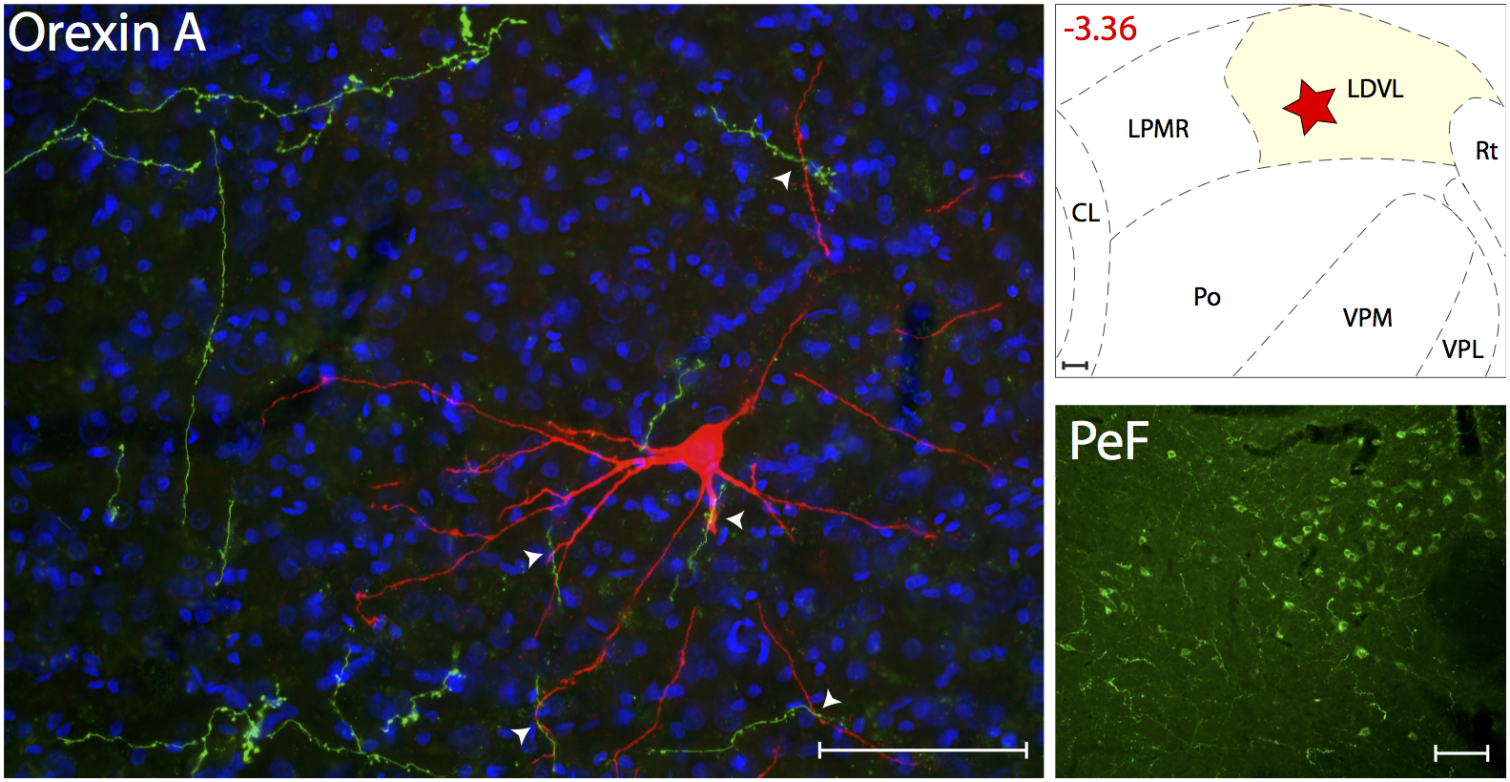
Orexinergic innervation of thalamic trigeminovascular neurons. Left: Immunopositive Orexin A axons (green) surrounding a thalamic dura-sensitive neuron (red) labeled with TMR–dextran. Nuclear counterstaining was performed with DAPI (blue). Arrowheads indicate close apposition of OrA positive axons and the dendrites of the labeled neuron. Upper right: Location of the dura-sensitive neuron (red star) shown at left. Number in red indicates distance from *bregma* (mm). Lower right: Fluorescent image showing OrA labeling of cell bodies in the hypothalamic perifornical area (PeF). Scale bars = 100 µm. Abbreviation: LDVL, laterodorsal thalamic, ventrolateral.

Orexinergic innervation was determined by targeting the neuropeptide orexin A (Fig. 10). Axons immunoreactive to orexin A were present at low density in LP, LD, Po and VPM. When examined in sections containing the trigeminovascular neuron(s), low density of orexinergic immunopositive axons and varicosities were seen in close apposition to the proximal and distal dendrites, but not the cell body (Fig. S7). These orexinergic axons originate mainly in the perifornical hypothalamic area.

### Thalamic trigeminovascular neurons are not innervated by CGRP, 5HT_1D_, oxytocin or vasopressin

**Figure 11:**
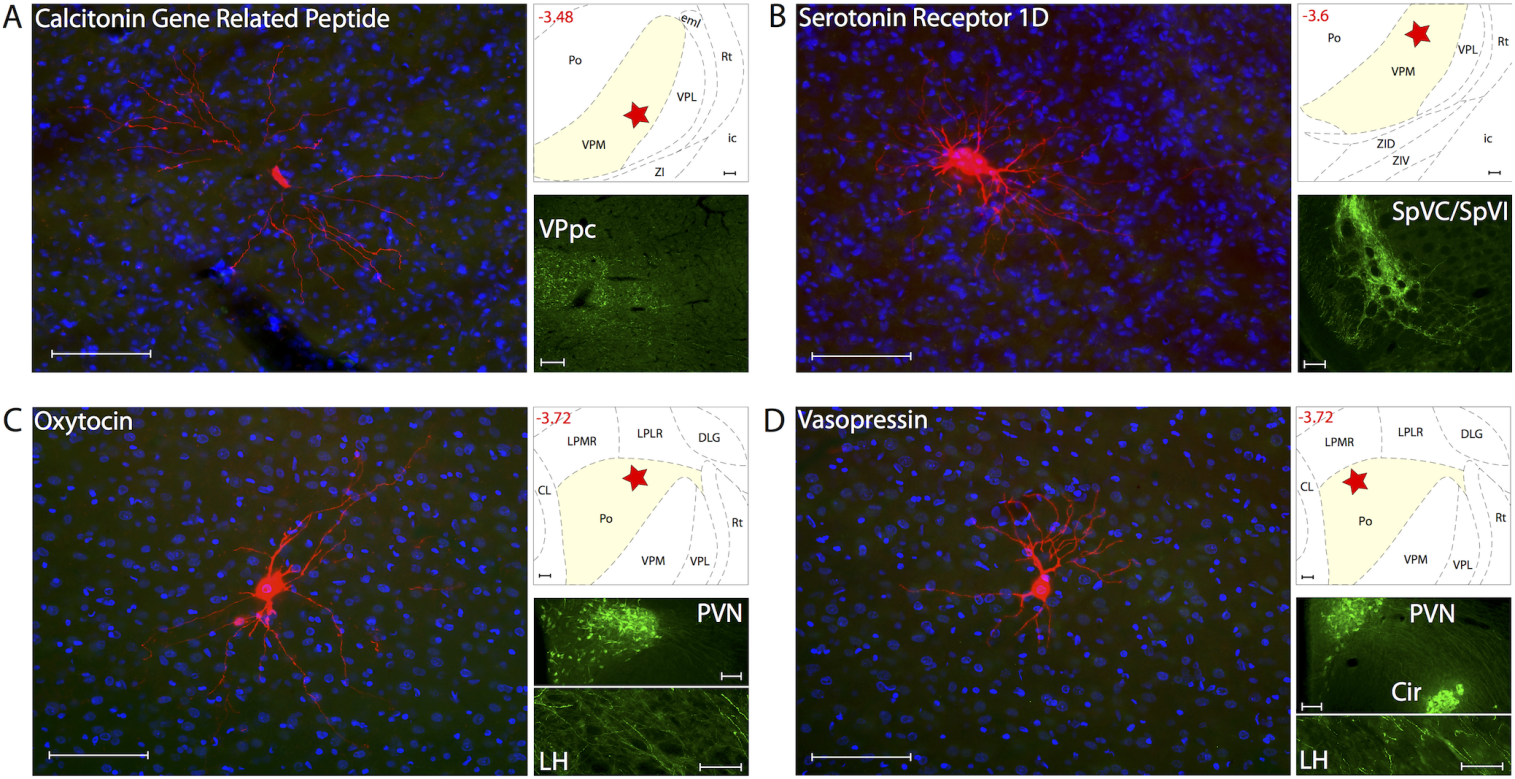
Lack of innervation of thalamic trigeminovascular neurons by axons containing CGRP, Serotonin 1D receptor, Oxytocin and Vasopressin. Left A–D: Thalamic dura-sensitive neurons (red) labeled with TMR–dextran and nuclear counterstain with DAPI (blue). Note the absence of axonal immunoreactivity to CGRP (A), Serotonin 1D receptor (B), Oxytocin (C) and Vasopressin (D). Upper right A–D: Locations of the dura-sensitive neurons (red stars) shown at left. Numbers in red indicate distance from *bregma* (mm). Lower right A–B: Fluorescent images showing CGRP (A) and Serotonin 1D receptor (B) immunopositive axons in the parvicellular division of the ventral posterior thalamic nucleus (VPpc) and the spinal trigeminal nuclei (SpVC/SpVI; caudal/interpolar), respectively. Lower right C: Fluorescent images showing Oxytocin labeling of cell bodies and axons in the hypothalamic paraventricular nucleus (PVN) and lateral hypothalamus (LH), respectively. Lower right D: Fluorescent images showing Vasopressin labeling of cell bodies in the PVN and circular (Cir) nuclei of the hypothalamus, and axons in the LH. Scale bars = 100 µm.

Surprisingly, we found no evidence for presence of CGRP-positive axons in any thalamic nucleus containing trigeminovascular neurons (positive identification of CGRP fibers in the parvicellular division of the ventral posterior thalamic nucleus confirms the validity of the negative staining in the thalamic nuclei analyzed in this study) (Fig. 11A). Similarly, we found no evidence for presence of 5HT_1D_ receptors in the relevant thalamic nuclei. Positive identification of 5HT_1D_ afferents in the medullary dorsal horn confirms the validity of the negative staining in the thalamic nuclei analyzed in this study (Fig. 11B). Predictably, we also found no evidence for innervation of thalamic trigeminovascular neurons by oxytocin or vasopressin from neurons in the paraventricular or supraoptic hypothalamic nuclei (Figs. 11C–D), demonstrating the selectivity of the positive findings.

### Density of thalamic innervation by the different biomarkers

We processed all images containing immunohistochemical evidence for thalamic innervation of the neurotransmitter/neuropeptides, and calculated their relative density by using binary maps (ImageJ). The binary map identifies all pixels containing positive immunostaining and converts them to white pixels; the remaining black pixels are considered lack of staining. This data provide quantitative measures of density of innervation of thalamic areas where juxtacellularly labeled trigeminovascular neurons were found (Table 1 and Figs. S8 and S9).

**Table 1:** Relative density of thalamic innervation by neurotransmitters and neuropeptides.

	Positive Pixels	Negative Pixels	Positive Pixels (%)	Density
**VGluT2**	139,571	1,308,109	**9.64**	High
**TH**	124,675	1,323,005	**8.61**	High
**VGaT**	106,398	1,341,282	**7.35**	High
**SERT**	82,190	1,365,490	**5.68**	High
**DBH**	46,331	1,401,349	**3.20**	Moderate
**Hist**	17,578	1,430,102	**1.21**	Moderate
**Orexin**	8,555	1,439,125	**0.59**	Low
**MCH**	7,153	1,440,527	**0.49**	Low
**CGRP**	0	1,447,680	**0.0**	Absent
**5HT1D**	0	1,447,680	**0.0**	Absent
**Vaso**	0	1,447,680	**0.0**	Absent
**Oxy**	0	1,447,680	**0.0**	Absent

Quantitative analysis using binary maps: >5% of positive (white) pixels per image indicates high density, 1–5% indicates moderate density, and <1% indicates low density of innervation. See Figs. S8 and S9 for actual binary maps.

## Discussion

**Figure 12:**
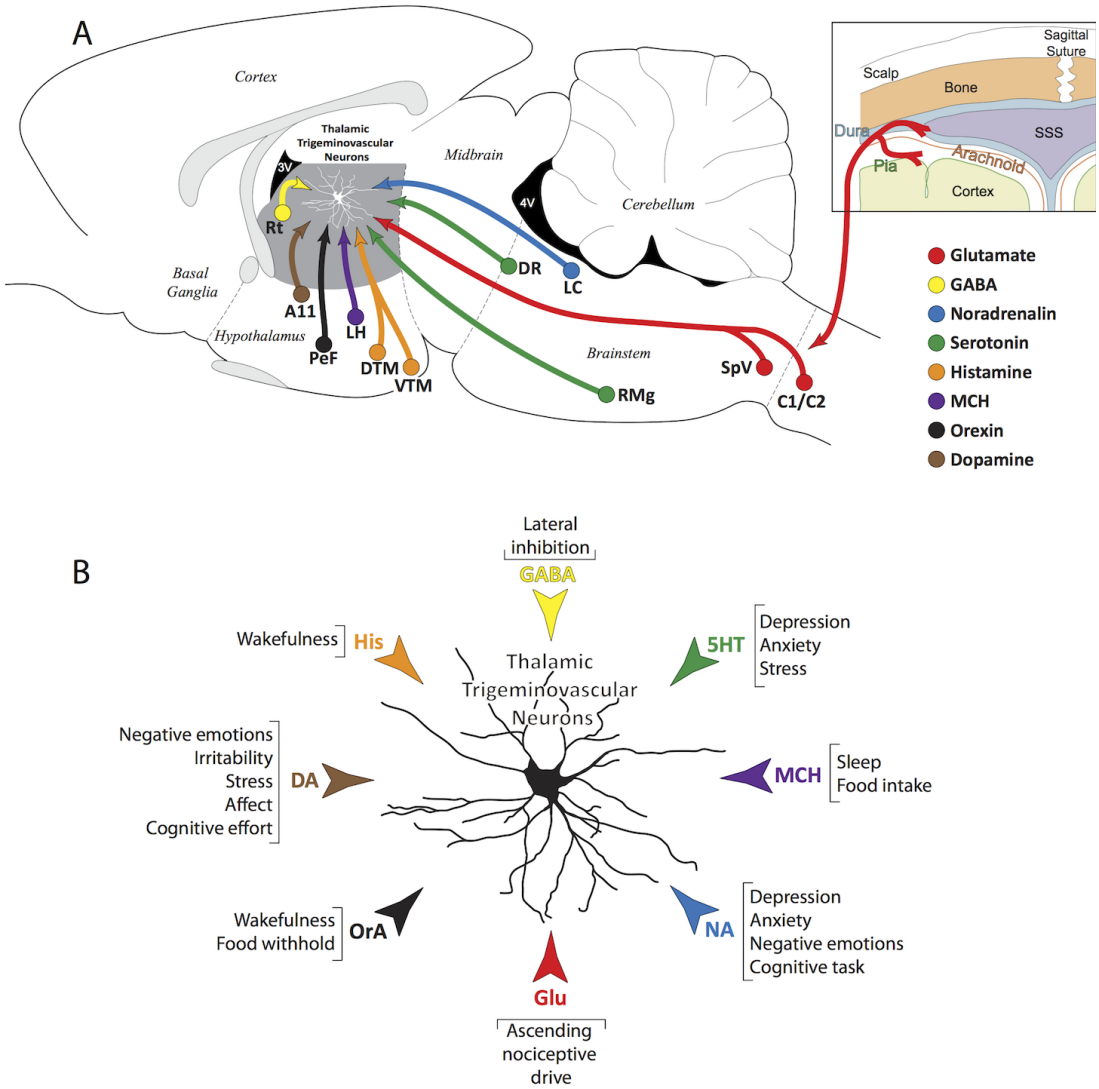
(A) Schematic illustration of the neurotransmitter and neuropeptidergic systems innervating thalamic trigeminovascular neurons in VPM, Po and LP/LD. The peripheral (meningeal nociceptors) and central (trigemino-thalamic) components of the trigeminovascular pathway are shown in red. The neurotransmitter and neuropeptidergic systems are color coded as follow: (a) Glutamate from SpVC/C1-2 in red; (b) GABA from Rt in yellow; (c) Noradrenalin from LC in blue; (d) Serotonin from raphe magnus (RMg) and dorsal raphe (DR) in green; (e) Histamine from DTM and VTM in orange; (f) Melanin Concentrating Hormone from LH in purple; (g) Orexin from PeF in black; (h) Dopamine from A11 in brown. (B) The diverse neurochemical pathways that converge on thalamic trigeminovascular neurons and the probability that many of them modulate neuronal activity in the same direction under certain conditions (e.g., sleep deprivation, wakefulness, food withhold, stress, anxiety) and in opposite directions under other conditions (e.g., food intake, sleep) define a sophisticated neuroanatomical network that may help us conceptualize how sensory, physiological, cognitive and affective conditions trigger, worsen or improve migraine headache.

This proof-of-concept study defines a new molecular framework for a more sophisticated thinking of the complexity of factors that modulate the response properties of relay trigeminovascular thalamic neurons. Most significant was the finding that such neurons receive direct input from axons containing glutamate, GABA, dopamine, noradrenaline, serotonin, histamine, orexin and MCH but not from axons that contain oxytocin, vasopressin, CGRP or the 5HT_1D_ receptor (Fig. 12A). This diverse input suggests that the transmission of headache-related nociceptive signals from the thalamus to the cortex is modulated by potentially opposing forces and that the so-called ‘decision’ of which system (neuropeptide/neurotransmitter) will dominate the firing of a trigeminovascular thalamic neuron at any given time is determined by the constantly changing physiological (sleep, wakefulness, food intake, body temperature, heart rate, blood pressure), behavioral (addiction, isolation), cognitive (attention, learning, memory use) and affective (stress, anxiety, depression, anger) adjustment needed to keep homeostasis (Fig. 12B).

The discharge mode of relay thalamocortical neurons is either burst or tonic [1], [29]. The burst discharge is commonly associated with lower excitability, drowsiness, and in the context of headache, responses to acute pain, whereas the tonic discharge has been associated with higher excitability, wakefulness, and chronic pain state [4], [30]–[32]. In principle, each of the neurotransmitters/neuropeptides found in this study to have close apposition with thalamic trigeminovascular neurons can potentially shift their firing mode from burst to tonic if it is excitatory, and from tonic to burst if it is inhibitory. As in other systems, the action of each neuropeptide/neurotransmitter on individual thalamic neuron depends on the type of release and reuptake, the type of receptor activated, and most likley the location of the neuron and its projection targets in the cortex. Since this information is not available for thalamic trigeminovascular neurons, which are the subject of this study, speculation on possible roles of the identified neuropeptides/neurotransmitters in setting thalamic transmission, as it may be related to migraine headache, is based on their known action in other systems.

### Glutamate

Vesicular glutamate transporters (VGluTs) are responsible for glutamate trafficking and for the subsequent regulated release of this excitatory neurotransmitter at the synapse. Glutamate excites relay thalamocortical neurons through NMDA receptors, if the sensory stimulus is prolong and through non-NMDA receptors if the sensory stimulus is brief [33], [34]. Of the three isoforms of VGluT, we opted to study VGluT2 because it is expressed most densely in relay thalamic nuclei [35]–[39] and in ascending trigeminal sensory neurons that project to VPM and Po [40], [41]. Since VGluT1 axons originate in corticothalamic neurons, we interpreted the presence of VGluT2 on thalamic trigeminovascular neurons as constituting the main drive for activation of these neurons by glutamatergic input they receive from ascending trigeminothalamic (possibly dura-sensitive) neurons in SpV.

### Dopamine

In the context of migraine, dopamine has been considered for its role in promoting hypothalamic-mediated symptoms/prodromes such as yawning and nausea [42], and more recently, modulation of dorsal horn trigeminovascular neurons [43]. Further supporting this hypothalamic connection is the finding that the A11 dopaminergic cell group in the medial hypothalamus innervates trigeminovascular neurons in both, the medullary dorsal horn [44], [45] and the thalamic relay nuclei [28]. The rich innervation of thalamic trigeminovascular neurons by TH-positive fibers suggests that modulation of transmission of nociceptive trigeminovascular signals by dopamine may also occur at the thalamus. When conceptualizing dopamine role in migraine, a consideration should be given to the activation of thalamic D_1_ and D_2_ receptors which facilitate membrane depolarization and increase spike discharge in somatosensory VPL/VPM thalamic neurons [46], and to the selective uptake of cocaine by dopaminergic nerve terminals in the thalamus as these findings define the possibility that thalamic dopamine pathways may be critically involved in drug-addiction, impulse control, affect, attention and decision making [47]–[53]. Translating these into clinical implications, thalamic dopamine may thus be considered as a possible contributor to behaviors that lead to medication-overuse headache and exacerbation of headache by negative emotions, effort to control anger and irritability, cognitive tasks that require attention and the need to make mundane decisions.

### Serotonin

Relevant to this study is that serotonin has long been implicated in migraine pathophysiology [54], [55], that this implication has lead to the development of 5HT_1B/1D_ receptor agonists (i.e., triptans) for acute treatment of migraine, that serotonergic innervation of VPM and Po originating mainly in the rostral raphe [9], [56]–[59], and that depending on the amount of serotonin release in the thalamus, it could be facilitatory (at low concentration) or inhibitory (at high concentration) to relay neurons in VPM and Po [60]. In principle, a high concentration of serotonin is inhibitory whereas a low concentration is excitatory. Accordingly, the very dense innervation of thalamic trigeminovasular neurons observed in our study can provide an anatomical substrate for a predominantly inhibitory effect of serotonin on transmission of trigeminovascular information between the thalamus and the cortex, as well as the inhibition of trigeminovascular thalamic neurons by local administration of 5HT_1_ agonists [61]. Given the latter, we were surprised by the total absence of 5HT_1D_ receptors in the thalamus. This finding suggests that the inhibition of thalamic trigeminovascular neurons response to dural stimulation occur at an earlier synapse along the trigeminovasculat pathway [62], rather than in the thalamus. On a more global view, serotonin, through its involvement in stress [63], anxiety [64], depression [65], sleep [66], apetite [67], and learning [64] may help facilitate the reciprocal relationship between these affective and physiological states and migraine.

### Noradrenaline

Because of the wide distribution of noradrenergic fibers in the brain it is difficult to assign to this neurotransmitter a specific role in certain function. Rather, it is thought to improve signal-to-noise ratio in the firing of neurons that respond to sensory stimuli [11], [68]–[70] when conditions involve anticipation, reward, and changing cognitive and emotional circumstances [71]. To be in a position to modulate thalamic neurons, noradrenergic fibers project heavily to all thalamic sensory nuclei [72], [73] and act on both α and β adrenoceptors, which together modulate firing rate, set a pacemaker current, determine membrane resting potential, and synaptic strength [74]–[77]. In the context of migraine, noradrenaline, which usually prolongs the activation of thalamic neurons [78]–[81], may be involved in setting abnormal excitability level in trigeminovascular neurons, centrally, and the magnitude of arterial hypertension, peripherally. This view is supported by the finding that β_1_ adrenoceptor blockers, which are among the very few drugs approved as migraine prophylactics [82], inhibit the activity of thalamic trigeminovascular neurons [83]. The observed relationship between noradrenergic fibers and thalamic trigeminovascular neurons provide a direct anatomical substrate for the central action of β_1_ adrenoceptor blockers in migraine. Given that activation of β_1_ adrenoceptor enhances the hyperpolarization-activated cation current (Ih) responsible for setting the so-called pacemaker activity level and the resting membrane potential in those relay thalamic neurons that exhibit such current [74], [76], [77], it is reasonable to speculate that thalamic trigeminovascular neurons exhibit the hyperpolarization-activated cation current – a current that may render them likely to exhibit a prolonged firing mode.

### Histamine

In the context of migraine, histamine has been considered for its role in causing H_1_ receptor mediated arterial dilatation and the consequential induction of delayed headache [84]. The findings that histaminergic nerve terminals converge on thalamic trigeminovascular neurons suggest that histamine role in migraine may also include modulation of thalamic trigeminovascular neurons through excitatory H_1_ receptors whose action enhances slow depolarization current capable of switching neuronal discharge mode from burst to tonic [85]. In the CNS, histamine originates exclusively from neurons of the tuberomammillary hypothalamic nucleus [17], [18]. Given that these neurons are active during the wake-state and quiescent during the sleep state [86]–[88] and that histamine switches the firing mode of relay thalamic neurons from burst to tonic [3], [85], it is tempting to speculate that the modulation of thalamic trigeminovascular neurons by the histaminergic pathway may play a role in the partial, or even complete, headache relief provided by sleep.

### Melanin Concentrating Hormone

The MCH system, which originates in the hypothalamus and contains GABA [89] is thought to play a modulatory/inhibitory role in the regulation of energy expenditure, arousal, locomotion, sexual behavior and a variety of autonomic functions [90]–[94]. Being excited by increased glucose level after a meal, MCH neurons are thought to promote sleep and energy expenditure (i.e., cessation of food intake) by releasing GABA at multiple cortical, subcortical, brainstem and spinal areas they project to. To date, this system has not been considered in the pathophysiology of migraine or other headaches. The findings that hypothalamic MCH neurons issue axons that terminate on thalamic trigeminovascular neurons define a novel anatomo-functional substrate for hypothesizing about possible interactions between food intake, drowsiness and migraine. It is tempting to propose that the mechanism by which eating may make patients ‘feel better’ during migraine involves increased level of glucose, activation of hypothalamic MCH neurons [95], and the consequential inhibition of relay thalamic trigeminovascular neurons. Conversely, this anatomo-functional substrate may also explain a part of the reasons for why migraine is promoted by skipping a meal. Skipping a meal inhibits MCH neurons (as glucose level goes down) that, when inactive, may release far less GABA around thalamic trigeminovascular neurons. Reduced GABA input might then enhance neuronal excitability, rendering them more likely to respond to subthreshold input they receive from ascending dura-sensitive neurons in the spinal trigeminal nucleus.

### Orexin

The orexin system originates in the lateral hypothalamus (LH) and projects to the cortex, thalamus, brainstem, spinal cord and other hypothalamic nuclei [20], [96]–[99]. It consists of 2 neuropeptides (orexin A, orexin B) that are synthesized by the same gene [100] and act on 2 classes of receptors, the selective orexin receptor 1 (orexin A) and the non-selective orexin receptor 2 (orexin A and B). The wide distribution of orexin fibers in the brain support a role in regulating food intake, arousal, wakefulness and sympathetically-mediated increase in body temperature, heart rate and blood pressure [101]. Opposite to the function of the MCH system, orexin neurons are excited by falling glucose levels, and their activation promotes food intake and wakefulness [102]–[104]. Of potential relevance to the pathophysiology of migraine are orexinergic axons in nociceptive laminae of the medullary dorsal horn and in close apposition to thalamic trigeminovascular neurons. Although no information is available regarding the direction in which orexin modulates thalamic trigeminovascular neurons, in vitro slice recording of thalamic neurons suggests that both orexin B and, for a lesser extent, orexin A are capable of depolarizing these neurons sufficently to switch their firing from the sleep-associated burst mode to the wakefulness-associated tonic mode [105]. In the context of migraine, it is thus reasonable to hypothesize that the mechanism by which eating may reduce headache intensity involves not only local release of GABA from activated MCH neurons but also inhibition of facilitatory orexin input to thalamic trigeminovascular neurons induced by increased glucose level (orexin neurons are inhibited by glucose). And conversely, fasting-induced fall in glucose activates the orexinergic neurons which in turn facilitate excitability through local release of orexin B and A.

### Calcitonin Gene Related Peptide

A large number of studies suggest that CGRP plays an important role in multiple aspects of migraine pathophysiolopgy [106]. Of particular relevance to the current study is the Summ et al., paper [107] demonstrating presence of CGRP receptors in VPM and consequently, inhibition of thalamic trigeminovascular neurons by systemic and local administration of CGRP receptor antagonists. The absence of CGRP-positive fibers in the vicinity of thalamic trigeminovascular neurons raises the possibility that CGRP release is not localized within the thalamus but rather may be released at a distant location of the receptors, acting as a neurohormone.

The thalamus is intricately connected with multiple cortical, subcortical and brainstem regions. It is viewed as an important subcortical hub with respect to functional brain networks [108] involved in processes that are altered in certain disease states [109], [110]. In the migraine brain, changes in modulation of thalamic neurons by various inputs may have significant effects on thalamic functional connectivity during both the interictal and the ictal state. The diverse neurochemical pathways that converge on thalamic trigeminovascular neurons (Fig. 12A–B) and the probability that many of them modulate neuronal activity in the same direction under certain conditions (e.g., sleep deprivation) and in opposite directions under other conditions (e.g., when satiated or scared) define a sophisticated neuroanatomical network that may help us conceptualize how sensory, physiological, cognitive and affective conditions trigger, worsen or improve migraine headache.

## Supporting Information

Figure S1 **Close apposition between VGluT2 immunopositive vesicles and thalamic trigeminovascular neurons.** The three views in the x–y, y–z and x–z planes provide evidence that VGluT2 immunopositive vesicles (green) may contact cell bodies, proximal and distal dendrites of trigeminovascular neurons in LP (red; as shown in Fig. 2). Arrowheads indicate probable contact point on each view. Note that some green-labeled vesicles and red-labeled soma or dendrites are in the same focal plane (yellow). Scale bar = 50 µm.
(TIFF)

Figure S2 **Close apposition between VGaT immunopositive vesicles and thalamic trigeminovascular neurons.** The three views in the x–y, y–z and x–z planes provide evidence that VGaT immunopositive vesicles (green) may contact cell bodies, proximal and distal dendrites of trigeminovascular neurons in VPM (red; as shown in Fig. 3). Arrowheads indicate probable contact point on each view. Note that some green-labeled vesicles and red-labeled soma or dendrites are in the same focal plane (yellow). Scale bar = 50 µm.
(TIFF)

Figure S3 **Close apposition between DBH immunopositive axons and thalamic trigeminovascular neurons.** The three views in the x–y, y–z and x–z planes provide evidence that DBH immunopositive fibers (green) may contact cell bodies, proximal and distal dendrites of trigeminovascular neurons in Po (red; as shown in Fig. 6). Arrowheads indicate probable contact point on each view. Note that some green-labeled axons and red-labeled soma or dendrites are in the same focal plane (yellow). Scale bar = 50 µm.
(TIFF)

Figure S4 **Close apposition between TH immunopositive axons and thalamic trigeminovascular neurons.** The three views in the x–y, y–z and x–z planes provide evidence that TH immunopositive fibers (green) may contact proximal and distal dendrites of trigeminovascular neurons in Po (red; as shown in Fig. 7). Arrowheads indicate probable contact point on each view. Note that some green-labeled axons and red-labeled dendrites are in the same focal plane (yellow). Scale bar = 50 µm.
(TIFF)

Figure S5 **Close apposition between Histamine immunopositive axons and thalamic trigeminovascular neurons.** The three views in the x–y, y–z and x–z planes provide evidence that Histamine immunopositive fibers (green) may contact cell bodies, proximal and distal dendrites of trigeminovascular neurons in LP (red; as shown in Fig. 8). Arrowheads indicate probable contact point on each view. Note that some green-labeled axons and red-labeled soma or dendrites are in the same focal plane (yellow). Scale bar = 50 µm.
(TIFF)

Figure S6 **Close apposition between MCH immunopositive axons and thalamic trigeminovascular neurons.** The three views in the x–y, y–z and x–z planes provide evidence that MCH immunopositive fibers (green) may contact distal dendrites of trigeminovascular neurons in VPM (red; as shown in Fig. 9). Arrowheads indicate probable contact point on each view. Note that some green-labeled axons and red-labeled dendrites are in the same focal plane (yellow). Scale bar = 50 µm.
(TIFF)

Figure S7 **Close apposition between Orexin A immunopositive axons and thalamic trigeminovascular neurons.** The three views in the x–y, y–z and x–z planes provide evidence that Orexin A immunopositive fibers (green) may contact proximal and distal dendrites of trigeminovascular neurons in LD (red; as shown in Fig. 10). Arrowheads indicate probable contact point on each view. Note that some green-labeled axons and red-labeled dendrites are in the same focal plane (yellow). Scale bar = 50 µm.
(TIFF)

Figure S8 **Density maps of thalamic innervation by neurotransmitters and neuropeptides.** Left: photomicrographs showing immunofluorescence staining of each biomarker in thalamic areas where juxtacellularly labeled trigeminovascular neurons were recorded (for anatomical reference, see figures 2–6). Right: Binary heat maps obtained from the images on the left showing all pixels (in red) containing positive immunostaining. Based on this data, objective measures to quantify density of innervation were obtained and defined as follow: High: >5% of positive pixels per image; Moderate: 1–5%; Low: <1%. The relative density of innervation by VGluT2, VGaT, SERT, DBH is 9.64% (high), 7.35% (high), 5.68% (high) and 3.2% (moderate) of positive pixels, respectively. Scale bar = 100 µm.
(TIFF)

Figure S9 **Density maps of thalamic innervation by neurotransmitters and neuropeptides.** Left: photomicrographs showing immunofluorescence staining of each biomarker in thalamic areas where juxtacellularly labeled trigeminovascular neurons were recorded (for anatomical reference, see figures 7–10). Right: Binary heat maps obtained from the images on the left showing all pixels (in red) containing positive immunostaining. The relative density of innervation by TH, Histamine, Orexin and MCH is 8.61% (high), 1.21% (moderate), 0.59% (low) and 0.49% (low) of positive pixels, respectively. Scale bar = 100 µm.
(TIFF)
