# Supplementary figures and images for: Neurochemical Pathways That Converge on Thalamic Trigeminovascular Neurons: Potential Substrate for Modulation of Migraine by Sleep, Food Intake, Stress and Anxiety
Source: PLoS One. 2014 Aug 4;9(8):e103929. doi: 10.1371/journal.pone.0103929 (PMC4121288; doi:10.1371/journal.pone.0103929)

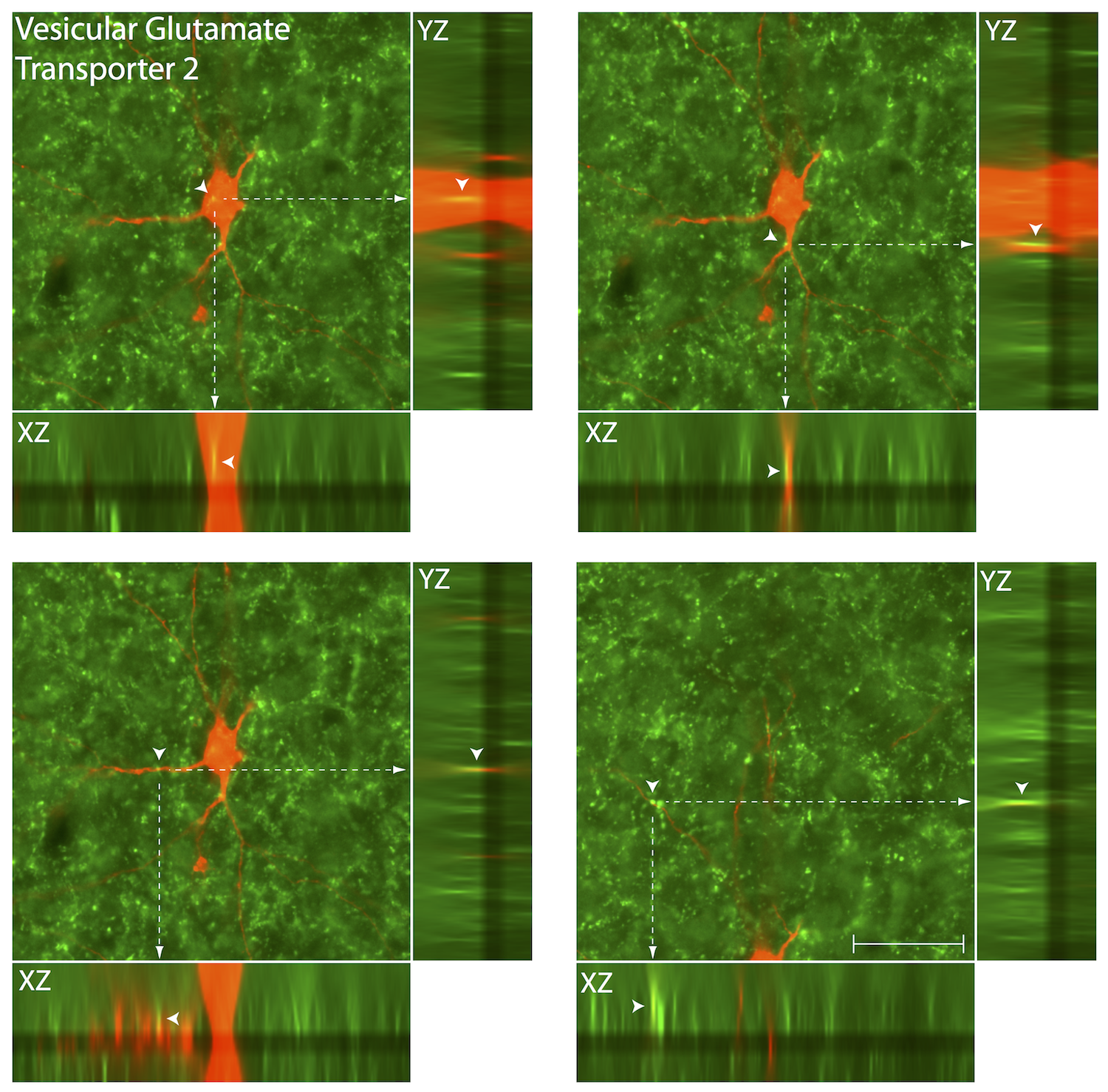

Supplement: Figure S1 — Close apposition between VGluT2 immunopositive vesicles and thalamic trigeminovascular neurons. The three views in the x–y, y–z and x–z planes provide evidence that VGluT2 immunopositive vesicles (green) may contact cell bodies, proximal and distal dendrites of trigeminovascular neurons in LP (red; as shown in Fig. 2). Arrowheads indicate probable contact point on each view. Note that some green-labeled vesicles and red-labeled soma or dendrites are in the same focal plane (yellow). Scale bar = 50 µm. (TIFF) [file pone.0103929.s001.tif]

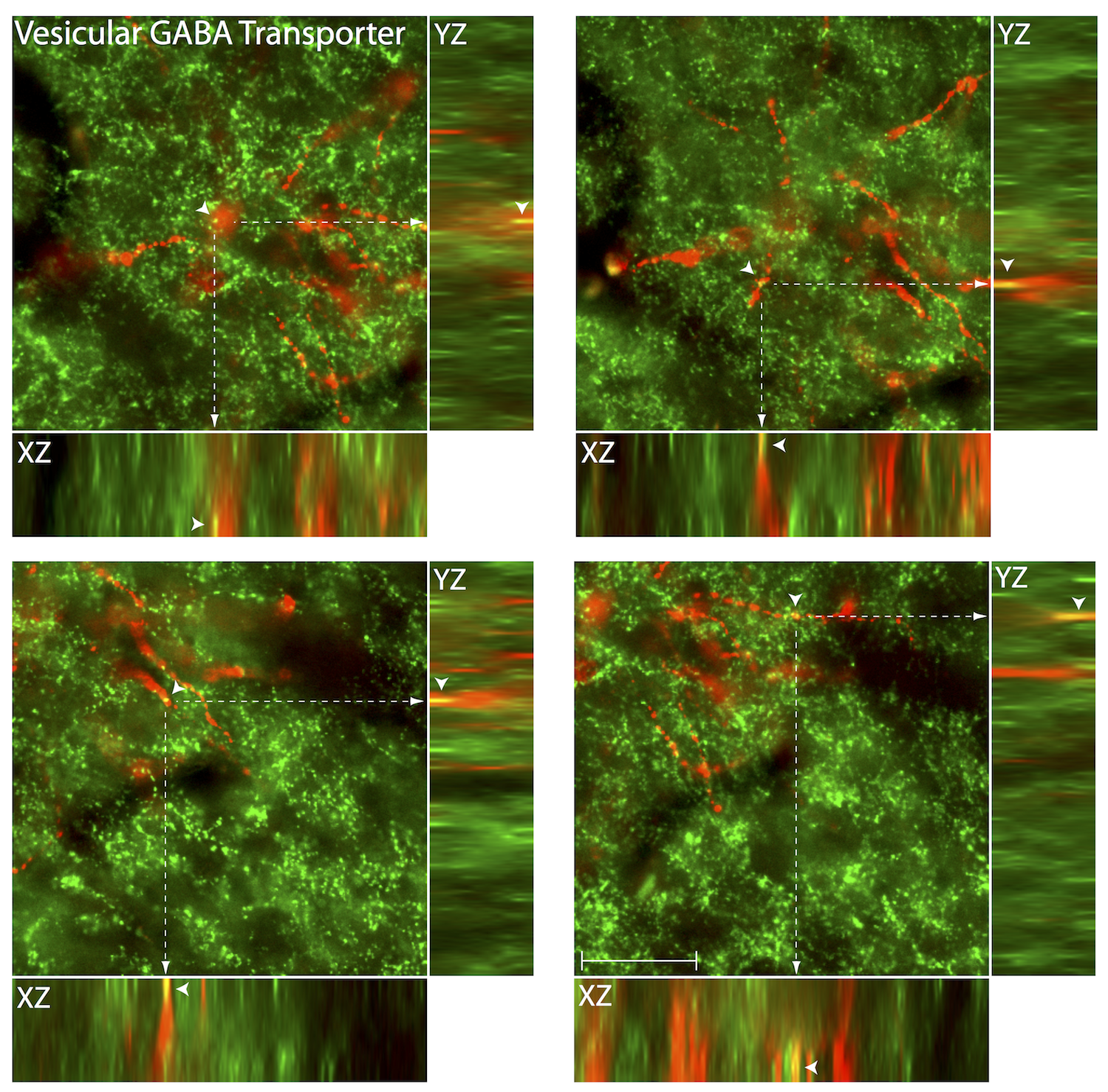

Supplement: Figure S2 — Close apposition between VGaT immunopositive vesicles and thalamic trigeminovascular neurons. The three views in the x–y, y–z and x–z planes provide evidence that VGaT immunopositive vesicles (green) may contact cell bodies, proximal and distal dendrites of trigeminovascular neurons in VPM (red; as shown in Fig. 3). Arrowheads indicate probable contact point on each view. Note that some green-labeled vesicles and red-labeled soma or dendrites are in the same focal plane (yellow). Scale bar = 50 µm. (TIFF) [file pone.0103929.s002.tif]

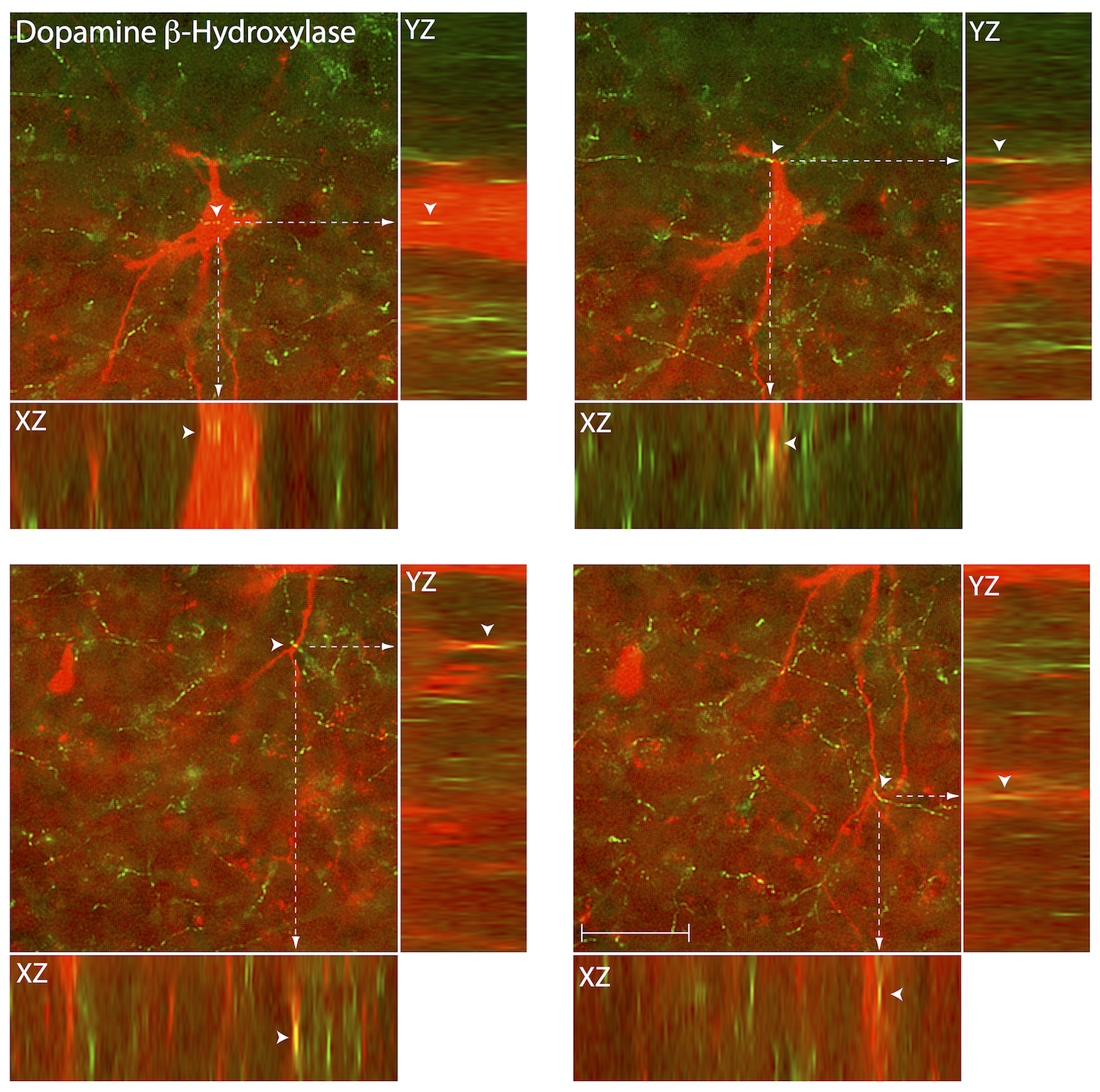

Supplement: Figure S3 — Close apposition between DBH immunopositive axons and thalamic trigeminovascular neurons. The three views in the x–y, y–z and x–z planes provide evidence that DBH immunopositive fibers (green) may contact cell bodies, proximal and distal dendrites of trigeminovascular neurons in Po (red; as shown in Fig. 6). Arrowheads indicate probable contact point on each view. Note that some green-labeled axons and red-labeled soma or dendrites are in the same focal plane (yellow). Scale bar = 50 µm. (TIFF) [file pone.0103929.s003.tif]

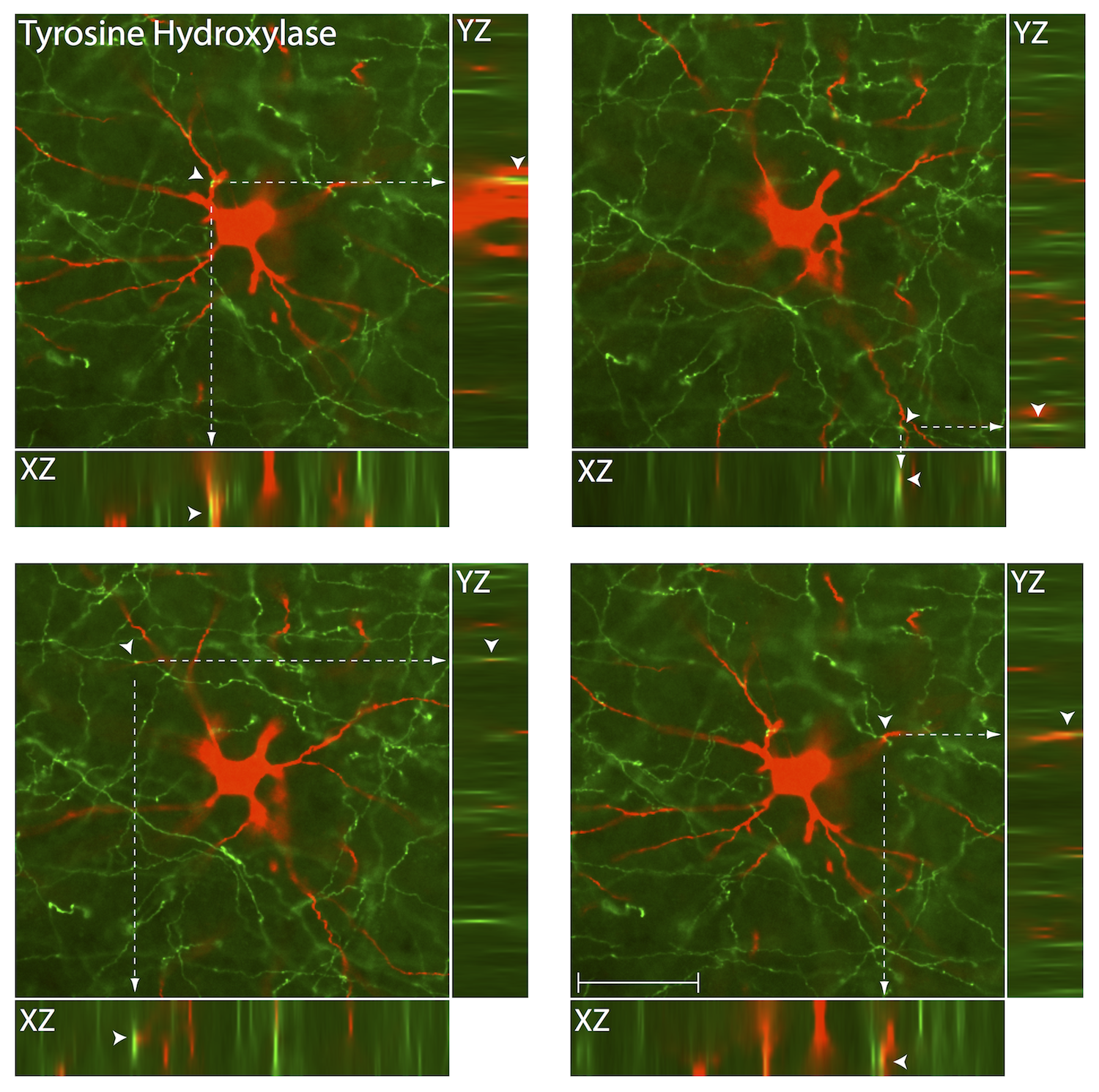

Supplement: Figure S4 — Close apposition between TH immunopositive axons and thalamic trigeminovascular neurons. The three views in the x–y, y–z and x–z planes provide evidence that TH immunopositive fibers (green) may contact proximal and distal dendrites of trigeminovascular neurons in Po (red; as shown in Fig. 7). Arrowheads indicate probable contact point on each view. Note that some green-labeled axons and red-labeled dendrites are in the same focal plane (yellow). Scale bar = 50 µm. (TIFF) [file pone.0103929.s004.tif]

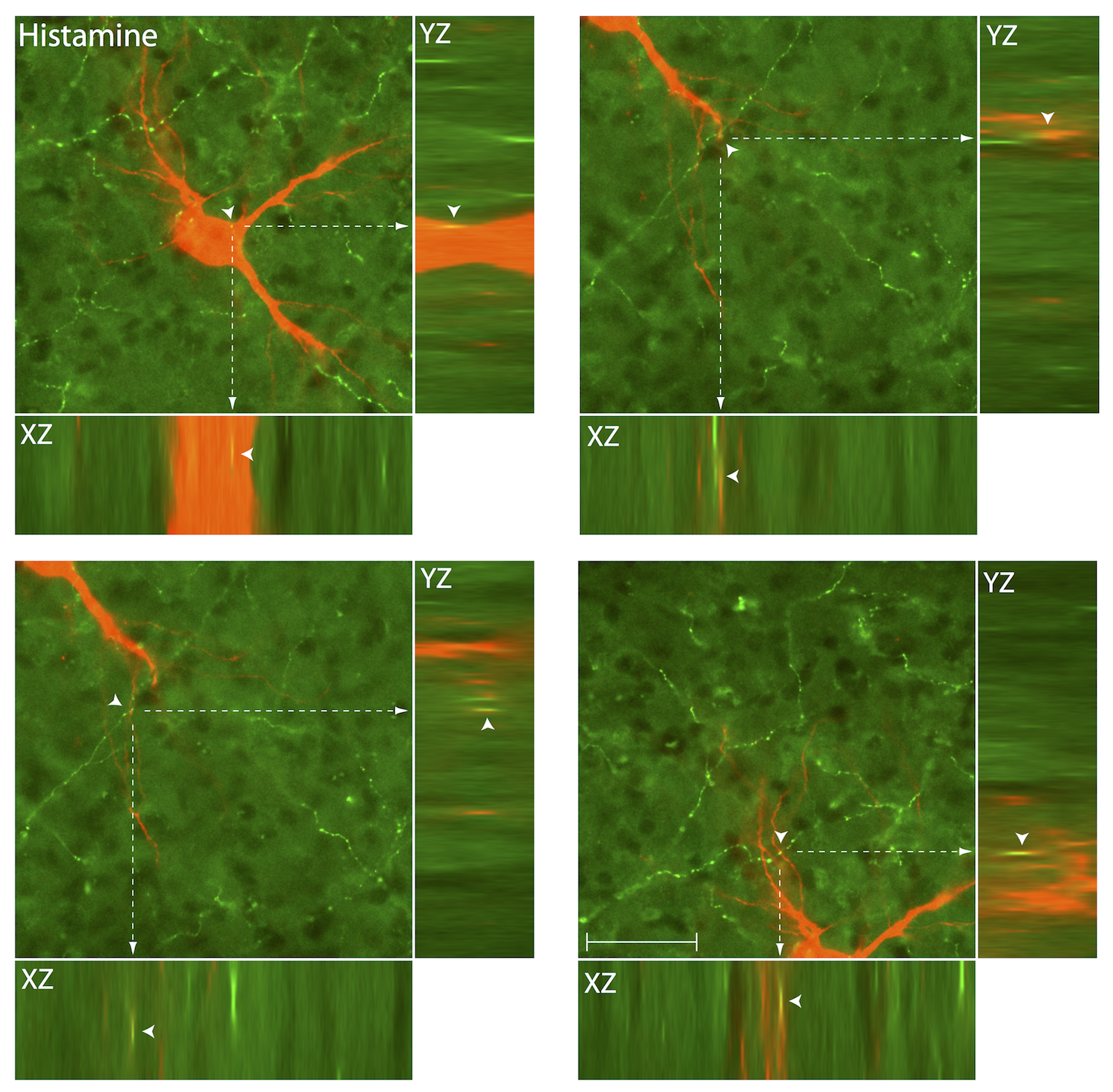

Supplement: Figure S5 — Close apposition between Histamine immunopositive axons and thalamic trigeminovascular neurons. The three views in the x–y, y–z and x–z planes provide evidence that Histamine immunopositive fibers (green) may contact cell bodies, proximal and distal dendrites of trigeminovascular neurons in LP (red; as shown in Fig. 8). Arrowheads indicate probable contact point on each view. Note that some green-labeled axons and red-labeled soma or dendrites are in the same focal plane (yellow). Scale bar = 50 µm. (TIFF) [file pone.0103929.s005.tif]

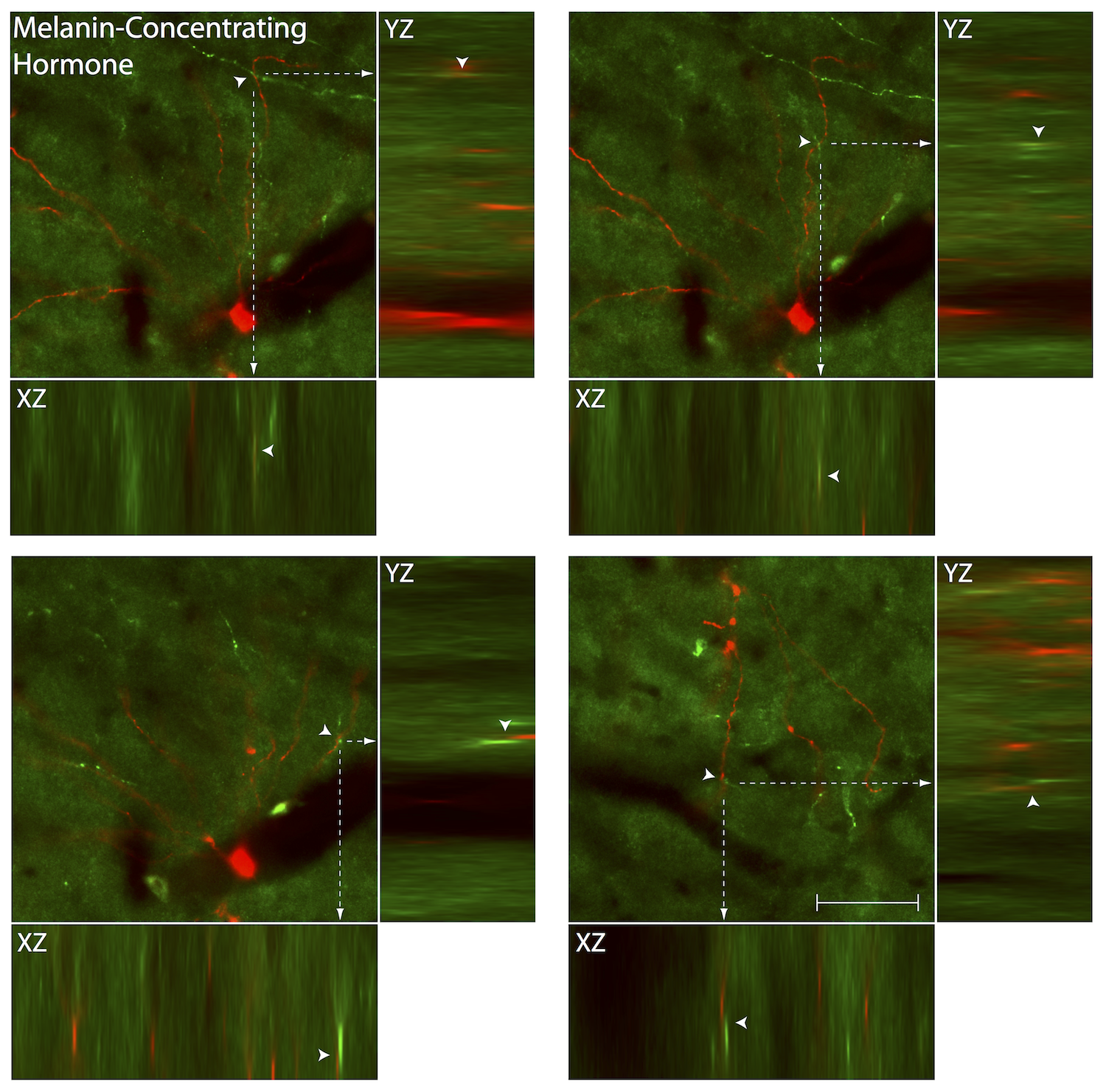

Supplement: Figure S6 — Close apposition between MCH immunopositive axons and thalamic trigeminovascular neurons. The three views in the x–y, y–z and x–z planes provide evidence that MCH immunopositive fibers (green) may contact distal dendrites of trigeminovascular neurons in VPM (red; as shown in Fig. 9). Arrowheads indicate probable contact point on each view. Note that some green-labeled axons and red-labeled dendrites are in the same focal plane (yellow). Scale bar = 50 µm. (TIFF) [file pone.0103929.s006.tif]

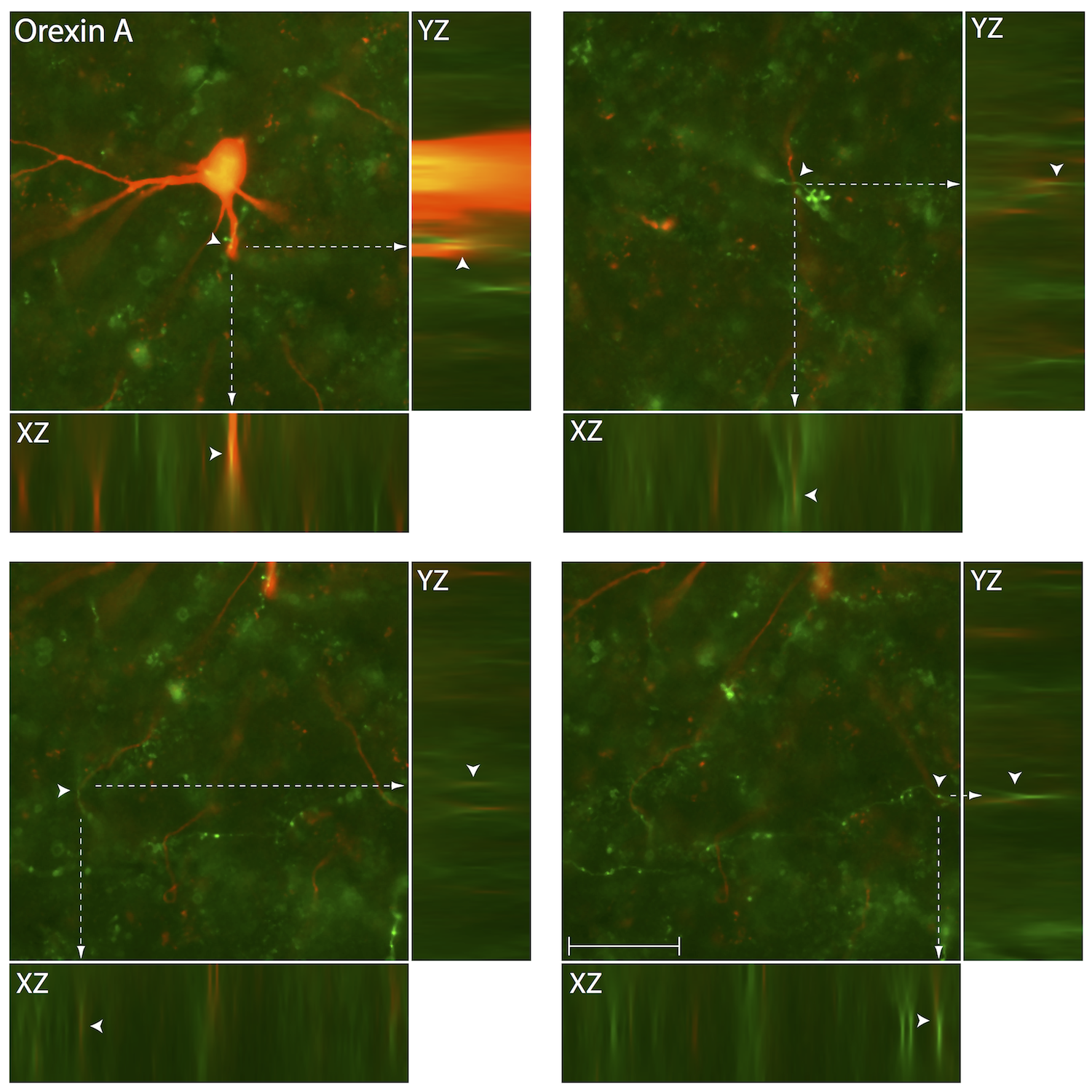

Supplement: Figure S7 — Close apposition between Orexin A immunopositive axons and thalamic trigeminovascular neurons. The three views in the x–y, y–z and x–z planes provide evidence that Orexin A immunopositive fibers (green) may contact proximal and distal dendrites of trigeminovascular neurons in LD (red; as shown in Fig. 10). Arrowheads indicate probable contact point on each view. Note that some green-labeled axons and red-labeled dendrites are in the same focal plane (yellow). Scale bar = 50 µm. (TIFF) [file pone.0103929.s007.tif]

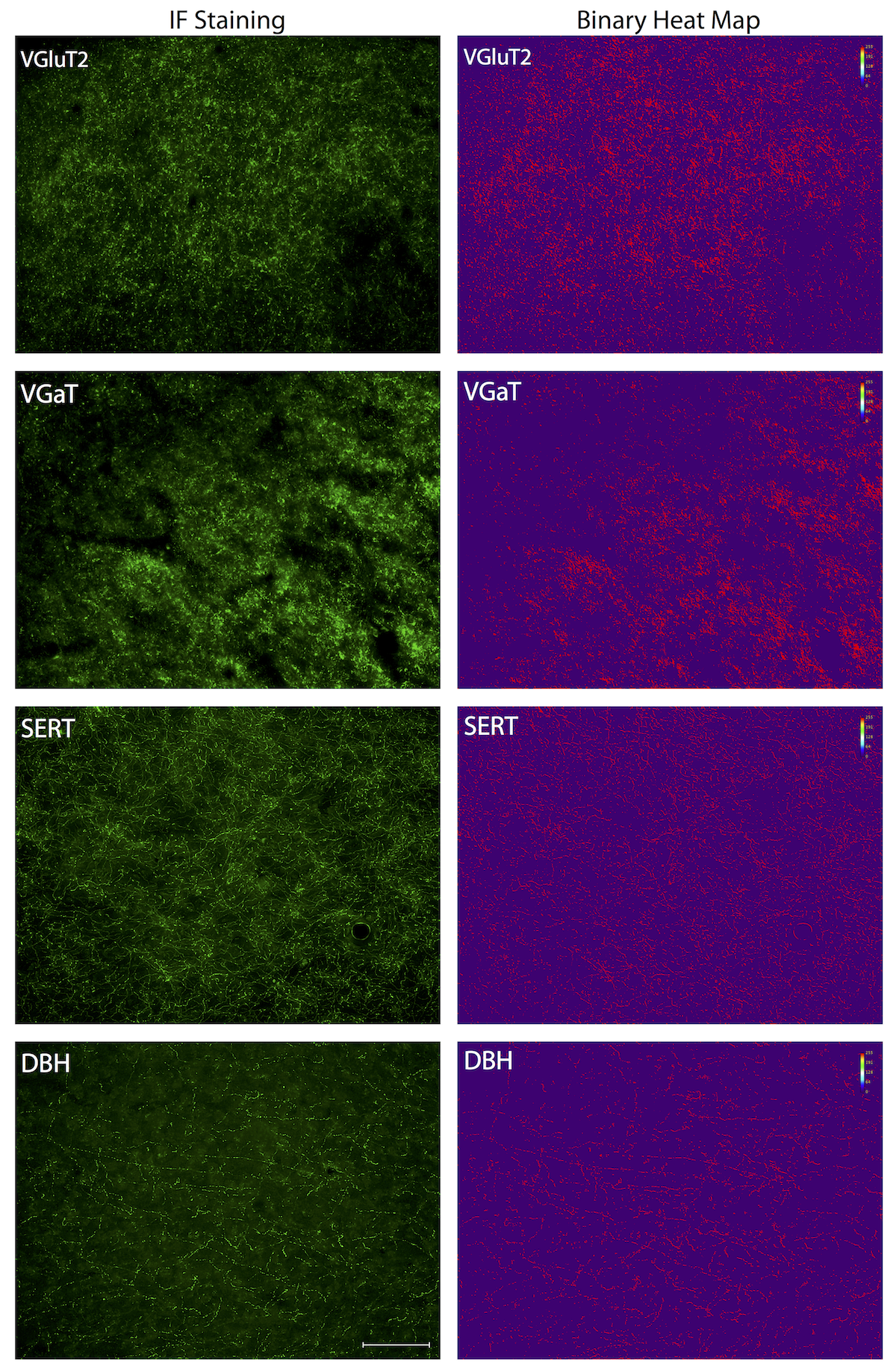

Supplement: Figure S8 — Density maps of thalamic innervation by neurotransmitters and neuropeptides. Left: photomicrographs showing immunofluorescence staining of each biomarker in thalamic areas where juxtacellularly labeled trigeminovascular neurons were recorded (for anatomical reference, see figures 2–6). Right: Binary heat maps obtained from the images on the left showing all pixels (in red) containing positive immunostaining. Based on this data, objective measures to quantify density of innervation were obtained and defined as follow: High: >5% of positive pixels per image; Moderate: 1–5%; Low: <1%. The relative density of innervation by VGluT2, VGaT, SERT, DBH is 9.64% (high), 7.35% (high), 5.68% (high) and 3.2% (moderate) of positive pixels, respectively. Scale bar = 100 µm. (TIFF) [file pone.0103929.s008.tif]

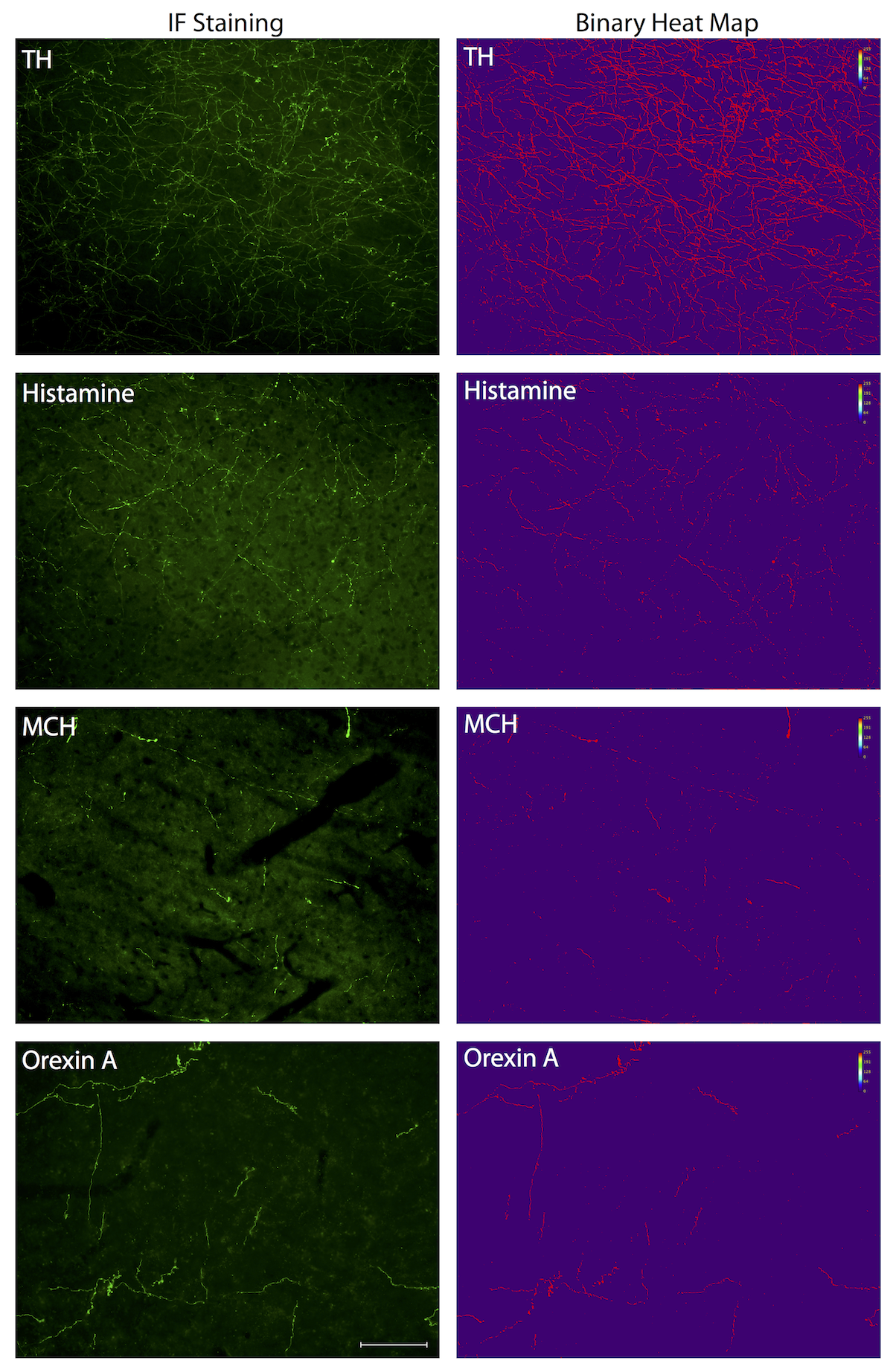

Supplement: Figure S9 — Density maps of thalamic innervation by neurotransmitters and neuropeptides. Left: photomicrographs showing immunofluorescence staining of each biomarker in thalamic areas where juxtacellularly labeled trigeminovascular neurons were recorded (for anatomical reference, see figures 7–10). Right: Binary heat maps obtained from the images on the left showing all pixels (in red) containing positive immunostaining. The relative density of innervation by TH, Histamine, Orexin and MCH is 8.61% (high), 1.21% (moderate), 0.59% (low) and 0.49% (low) of positive pixels, respectively. Scale bar = 100 µm. (TIFF) [file pone.0103929.s009.tif]
